# Agentic EEG Pipeline Synthesis Using Large Language Models and the Model Context Protocol

**DOI:** 10.3390/s26206472

**Published:** 2026-10-13

**Authors:** Kylie K. Higashionna, Jasmine M. Meissner, Katy Tarrit

**Affiliations:** 1 Department of Information and Computer Sciences, University of Hawai‘i at Mānoa, Honolulu, HI 96822, USA; 2 College of Engineering, University of Hawai‘i at Mānoa, Honolulu, HI 96822, USA; jmeissne@hawaii.edu

**Keywords:** electroencephalography, EEG processing, brain–computer interface, large language model, Model Context Protocol, agentic AI, signal processing, EEGLAB

## Abstract

Electroencephalography analysis typically requires multistage processing pipelines for which configuration depends on recording characteristics and the intended analytical objective. Although automated pipelines improve reproducibility, they generally implement predefined processing sequences. We present an agentic framework in which a large language model (LLM) orchestrator synthesizes and executes task-specific EEG processing workflows by selecting, parameterizing, and ordering established signal processing components accessed through Model Context Protocol interfaces. Computational EEG processing and analysis operations are performed using established EEGLAB (version 2026.0.0) implementations running in MATLAB (R2026a), while structured execution results and quality control information are returned to the orchestrator to support adaptation during execution. The framework is evaluated by examining tool-layer execution fidelity, independent pipeline synthesis, and the quality of the resulting EEG output relative to the established DISCOVER-EEG workflow. In an evaluation using eight resting state EEG recordings from four participants, mean epoch retention was 0.845 for the agent-generated pipeline and 0.843 for DISCOVER-EEG, while mean spectral entropy was 4.87 and 4.84 bits, respectively. Mean signal-to-noise ratio was 13.92 dB for the agent-generated pipeline and 11.14 dB for DISCOVER-EEG; however, this difference should be interpreted cautiously because the pipelines used different upper filter boundaries. A secondary analysis examined whether EEG processed using the generated pipeline retained features relevant to Alzheimer’s disease versus healthy control classification. These findings demonstrate the feasibility of coupling LLM-based orchestration with EEG processing software to generate executable task-specific workflows with structured execution records and provenance. Further evaluation across larger datasets, independent synthesis runs, and additional EEG paradigms is required in order to establish repeatability and generalizability.

## 1. Introduction

Electroencephalography (EEG) is a widely used neurophysiological recording technique in brain–computer interfaces (BCIs), neurofeedback, cognitive neuroscience, and clinical neurological assessment. It provides non-invasive measurements of cortical electrophysiological activity with millisecond-scale temporal resolution and relatively low acquisition cost compared to many neuroimaging modalities [1]. Scalp-recorded EEG reflects the superposition of neural activity across multiple spatial and temporal scales, and contains physiologically relevant information in both ongoing neural oscillations and event-related responses. However, these signals are also affected by non-neural activity and acquisition-related interference, including ocular and myogenic artifacts, movement-related artifacts, line noise, and poorly coupled or malfunctioning electrodes. Extracting reliable neurophysiological features from raw EEG requires careful preprocessing to enhance signal quality while preserving neural activity relevant to the intended analysis. Decisions involving temporal and spectral filtering, re-referencing, bad channel detection and interpolation, artifact rejection or correction, epoching, and feature extraction can directly influence the morphology, spectral content, and spatial distribution of the resulting EEG signals. Consequently, the validity and reproducibility of EEG-derived measures depend not only on the sensing hardware and downstream analytical model but also on the signal processing procedures used to transform the acquired electrophysiological recordings into interpretable neural features [2].

Processing choices also vary with the neurophysiological phenomenon being examined and the intended downstream analysis. For example, resting state EEG studies commonly characterize oscillatory activity within frequency bands such as delta, theta, alpha, and beta, whereas event-related potential analyses emphasize the preservation of stimulus-locked waveform morphology, amplitude, and latency [3,4,5,6,7]. Motor imagery BCIs may rely on sensorimotor rhythm modulation and event-related desynchronization or synchronization [8,9], while functional connectivity analyses require particular attention to filtering, referencing, volume conduction, and artifact contamination [10,11]. Accordingly, preprocessing choices such as filter boundaries, reference scheme, epoch duration, artifact removal criteria, and data rejection thresholds may need to be adapted across EEG applications. These choices are further constrained by acquisition characteristics, including the sampling frequency, electrode montage and density, recording duration, mains frequency, signal quality, and prevalence and type of physiological and non-physiological artifacts. Processing operations are also interdependent; filtering can influence independent component analysis (ICA), while channel rejection and interpolation can affect subsequent re-referencing and scalp representations. At the same time, artifact correction and epoch rejection criteria can alter the characteristics and amount of data retained for analysis [3,4]. As such, EEG preprocessing requires a coordinated set of signal processing decisions that balances artifact attenuation, preservation of relevant neural activity, and the requirements of the intended downstream analysis.

Several approaches have been developed to automate EEG preprocessing and improve the reproducibility of electrophysiological analyses. The PREP pipeline standardizes early-stage preprocessing operations, including line noise removal, bad channel detection, and robust referencing [12]. HAPPE provides an automated preprocessing framework designed particularly for developmental and other EEG datasets with high artifact burden [13]. Automagic integrates established preprocessing procedures with automated quality assessment and reporting [14], while DISCOVER-EEG provides a standardized automated workflow for resting state EEG preprocessing, feature extraction, and visualization [15]. Collectively, these frameworks reduce the need for manual intervention while promoting consistency and reproducibility across EEG analyses. However, these approaches generally operate within predefined processing architectures, even when individual operations or parameters are adapted to characteristics of the data; consequently, adapting an existing pipeline to a different acquisition setting, neurophysiological signal of interest, or downstream analytical objective may require manual modification of processing steps and parameters. This creates a need for approaches that can dynamically select, parameterize, and organize EEG processing operations according to the characteristics of the acquired signals, experimental paradigm, recording environment, and requirements of the intended analysis while still maintaining a clear and reproducible processing workflow.

To address this need and enable the automated generation of task-specific EEG processing pipelines, we propose and evaluate an agentic framework that translates a user-defined EEG analysis objective into a fully specified and executable EEG processing workflow. The framework considers the user-defined analysis objective together with EEG recording metadata to select, parameterize, and organize processing operations in a task- and data-informed manner. Our approach uses the Model Context Protocol (MCP) to provide a large language model (LLM)-based orchestrator with structured access to EEG processing operations, enabling it to discover available tools, invoke them with explicit parameters, and interpret the processing summaries and signal quality information returned during execution. EEGLAB-based operations such as filtering, line noise removal, bad channel detection and interpolation, artifact subspace reconstruction (ASR), ICA with ICLabel-based component classification, epoching, and spectral feature extraction are made available to the orchestrator through structured composable tools. Importantly, the LLM does not generate or implement the underlying numerical signal processing algorithms; it uses established implementations to determine which operations to apply, how to parameterize and order them, and whether the resulting EEG data remain compatible with the requirements of the intended analysis. This process produces an executable and reproducible EEG processing pipeline. During execution, intermediate processing summaries and signal quality measures are returned to the orchestrator, allowing it to assess processing outcomes and revise the workflow when additional or modified steps may be warranted. Each tool invocation, parameter selection, intermediate result, and quality control measure is recorded, creating a transparent provenance trail that supports inspection and reproducibility. The framework extends conventional EEG processing automation beyond predefined workflows towards a task-directed pipeline synthesis in which the workflow is generated according to the intended analysis, acquired signal characteristics, and user-defined requirements. This capacity for quality control-driven revision is supported by the architecture, but was not systematically evaluated in the present study.

We evaluate the proposed framework in three experiments. First, we assess tool-layer execution fidelity by implementing the established DISCOVER-EEG workflow through the MCP tool interface and comparing the resulting outputs with those produced by the original implementation; this evaluation is designed to distinguish implementation-level discrepancies in the tool-mediated execution layer from differences arising from agent-generated processing decisions. Second, we allow the agent to independently synthesize an EEG processing workflow and compare its performance with the reference pipeline using eyes-open and eyes-closed resting state EEG recordings; this comparison evaluates data retention, signal quality, and spectral features to determine whether the generated workflow preserves task-relevant EEG information while attenuating noise and artifacts. Third, we investigate whether the generated workflow can support a downstream clinical application involving EEG-derived biomarkers for distinguishing individuals with Alzheimer’s disease from healthy controls [16,17,18,19,20,21,22]. Together, these experiments evaluate the execution fidelity of the tool-mediated framework and the feasibility of task-directed agentic reasoning for synthesizing executable EEG processing workflows.

The principal contributions of this study are as follows:

- An agentic EEG processing framework that generates fully executable task-specific processing pipelines based on user-defined analysis objectives and characteristics of the acquired EEG data.
- A structured architecture that connects LLM-based reasoning to established EEGLAB signal processing operations through MCP-based tool interfaces.
- A validation strategy that separately evaluates tool-layer execution fidelity and the performance of agent-generated EEG processing pipelines.
- Empirical evaluation against DISCOVER-EEG based on data retention, signal quality, and spectral features, together with a downstream EEG biomarker analysis for Alzheimer’s disease classification.

## 2. Related Work

Recent studies have investigated the use of large language models and agentic architectures to support EEG analysis, automate multistage processing, and increase the accessibility of electrophysiological workflows.

BrainAgent [23] is a hierarchical and extensible multi-agent framework designed to automate end-to-end brain signal analysis. An LLM-supervisor agent, the orchestrator, is given the user query and raw EEG data; it then decides whether to decompose the query into a structured plan and delegate sub-tasks to specialized agents. The specialized agents receive only the task context required for their assigned operations, with the aim of constraining scope and reducing unnecessary reasoning or tool use. Once the tasks of the sub-agents are completed, the orchestrator synthesizes the intermediate reports of the sub-agents and reasons to generate a medically coherent analysis. The reported evaluation focused primarily on publicly available sleep datasets, leaving broader generalization across EEG paradigms and processing objectives for future study.

Xu and Dai proposed NeuraDock, an open-source architecture that separates a deterministic local EEG processing engine from a hardware-aware language model interface [24]. The system first parses the recordings and produces machine-readable artifacts before providing the language model with a compact summary and versioned context representation. The reported implementation focused on low-channel-count EEG and emphasized boundary-aware handling of negative and ambiguous findings.

EEGAgent [25] is a similar approach that uses LLMs to process EEG data. The agent first utilizes clinical literature along with metadata to form an initial expectation of EEG patterns before using parametric and non-parametric EEG processing tools to conduct the analysis. When tested on seizure data, it was able to pinpoint temporal and spatial regions that exhibited key biomarkers for seizures. Although the framework achieved a reported hit rate of 69.30% at an intersection-over-union threshold of 0.7, a false positive rate of 44.77% was also reported.

Similarly, several prior studies have explored transformer-based architectures for modeling the temporal structure of EEG signals [26,27,28,29,30]. These approaches typically tokenize segments of raw or minimally preprocessed EEG recordings for input to transformer-based models, in some cases incorporating associated metadata. Although such EEG foundation models can support a range of downstream tasks, they generally require substantial pretraining datasets followed by task-specific adaptation or fine-tuning. Importantly, these approaches focus on learning representations directly from EEG signals, whereas our framework uses an LLM to orchestrate the selection, parameterization, and execution of EEG processing operations.

The use of agentic frameworks for engineering and scientific domains is not limited to EEG signal processing, also finding use in a variety of applications. AutoSizer is a two-loop framework for optimizing analog and mixed-signal integrated circuits that reduces the expert knowledge required to handle circuit design constraints such as transistor sizing, design spaces, and performance limitations [31]. The framework uses multiple agents across different stages of the circuit optimization process, beginning with circuit understanding and search space definition to determine which variables and ranges should be prioritized. To execute circuit sizing optimization, a tailored optimization agent is equipped with a variety of tools and simulation result checks before deciding whether the objective has been satisfied or whether to reevaluate the search space based on the convergence analysis and impact on the variables.

In a similar vein, Bran et al. and Campbell et al. equipped large language models with chain-of-thought reasoning and expert-designed tools to aid in molecular modeling and the discovery of new compounds. Bran et al.’s ChemCrow [32] utilizes a reasoning loop consisting of thought, action, action input, and observation to reason and plan out which tool to select before using the tool and analyzing the output; it then takes into account molecule, safety, reaction, and general search tools. By contrast, Campbell et al.’s MDCrow [33] automates molecular dynamics workflows using an iterative think–act–observe loop to complete each subtask in a user prompt. It draws on four categories of tools: information retrieval, file and protein handling, simulation, and analysis.

These works demonstrate that agentic LLM-based frameworks can make highly technical knowledge more easily available, lowering barriers to research and discovery; by applying these ideas to EEG analysis, the framework in this paper aims to automate signal processing techniques so that they are more accessible to clinicians and researchers.

In contrast to systems that primarily perform interpretation, task-specific analysis, or fixed tool delegation, the present framework focuses on explicit synthesis of an executable EEG processing pipeline from a reusable catalog of validated operations. The central question is not whether an LLM can directly interpret EEG signals but whether it can select, parameterize, and order established signal processing components and adapt processing decisions in response to returned execution information.

## 3. Materials and Methods

### 3.1. Approach Overview and Problem Formulation

This study investigates whether an agentic EEG pipeline synthesis approach can rely on a user-defined analysis objective together with characteristics of an input EEG dataset to construct a fully executable task- and data-compatible EEG processing pipeline.

The framework provides the agent with a catalog of EEG processing components implemented using EEGLAB [34] and MATLAB [35]. Rather than implementing numerical signal processing algorithms itself, the agent selects components from the available catalog, configures their parameters, determines their execution order, and composes them into a task-specific processing workflow.

We formulate the pipeline synthesis problem using four elements: a user-defined EEG analysis task *q*, an input EEG dataset and the dataset information available to the orchestrator *X*, a catalog of available EEG processing components C, and an agentic pipeline generator *G*. The component catalog contains EEG processing tools, functions, and scripts implemented using EEGLAB (version 2026.0.0) and MATLAB (R2026a). Given the analysis task, dataset characteristics, and available components, pipeline generation is expressed as

(1) $$P=G\left(q,X,C\right),$$

where *P* denotes the resulting executable EEG processing pipeline.

The generated pipeline is represented as an ordered sequence of parameterized component invocations:

(2) $$P=\langle c_{1}\left(\theta_{1}\right),c_{2}\left(\theta_{2}\right),\ldots ,c_{n}\left(\theta_{n}\right)\rangle ,\;c_{i}\in C,$$

where $c_{i}$ denotes an EEG processing component selected from the catalog and $\theta_{i}$ denotes the parameter configuration assigned to that component by the orchestrator. The resulting pipeline synthesis involves three related decisions: selecting the components to include, determining a valid execution order, and configuring the parameters of each selected component.

The task *q* specifies the intended analysis objective, such as “preparing resting state EEG data for spectral analysis” or “extracting EEG-derived features for downstream clinical reasoning”. The dataset *X* includes the EEG recordings together with available information about their acquisition and organization, including sampling frequency, channel montage, recording duration, event structure, recording condition, and BIDS metadata. These characteristics provide context for processing decisions such as resampling frequency, line noise removal, filter boundaries, epoch construction, artifact handling, and feature extraction.

The framework synthesizes pipelines only from components available in C. It does not ask the language model to implement filtering, independent component analysis (ICA), artifact correction, or other numerical signal-processing algorithms. Instead, the agent reasons over established implementations and produces a machine-executable composition of those operations. This separation allows agentic reasoning to be used for pipeline construction while numerical EEG processing remains delegated to validated scientific software. Accordingly, the space of pipelines that can be synthesized is constrained by the processing operations and parameter configurations exposed through the component catalog.

### 3.2. Agentic EEG Pipeline Synthesis Architecture

**Figure 1:**
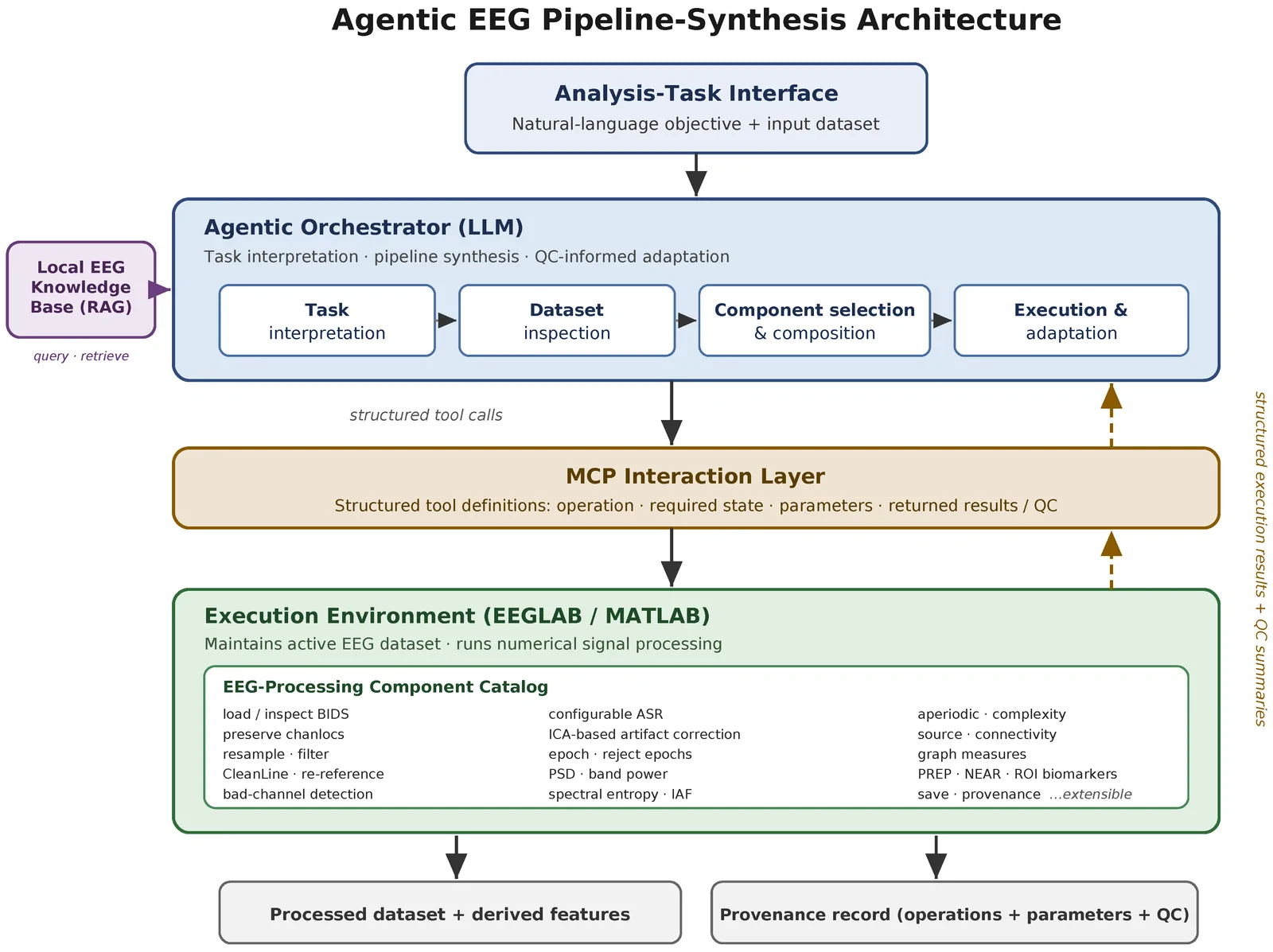
Architecture of the agentic EEG pipeline synthesis framework. A natural language analysis task and input dataset enter through the analysis task interface. The agentic orchestrator performs task interpretation, dataset inspection, component selection and composition, and execution with adaptation, issuing structured tool calls through the MCP interaction layer to the EEGLAB/MATLAB execution environment, which runs the numerical signal processing drawn from the EEG processing component catalog. Execution results and quality control summaries are returned to the orchestrator to inform subsequent decisions. The workflow yields the processed dataset with derived features and a provenance record of the executed operations and parameters.

The framework consists of five principal elements: the analysis task interface, the agentic orchestrator, the EEG processing component catalog, the MCP interaction layer, and the EEG execution environment. Figure 1 presents the overall architecture.

- The **analysis task interface** receives a description of the user’s intended EEG analysis in natural language. The task description identifies the expected analytical outcome, such as “preparing resting state EEG recordings for spectral and connectivity analysis” or “performing a clinically informed EEG evaluation of a patient undergoing cognitive assessment”. The interface also identifies the input dataset to be processed.
- The **agentic orchestrator** interprets the requested task, inspects the available dataset information, discovers the processing capabilities exposed by the system, and constructs the pipeline. It selects processing operations from the available catalog, determines their dependencies and execution order, and configures their parameters. During execution, the orchestrator also receives intermediate processing summaries and quality control information returned by the processing components. These outputs are incorporated into the active processing context, and can be used to revise processing decisions or parameter settings when the returned results indicate that additional or modified processing may be warranted. In this way, the orchestrator manages both pipeline synthesis and adaptive execution across successive processing stages.
- The **EEG processing component catalog** contains reusable signal processing capabilities implemented using EEGLAB and MATLAB. Each component performs a bounded operation, such as applying a filter, detecting bad channels, computing an ICA decomposition, correcting artifacts, or extracting spectral features. Separating these capabilities into individually callable components permits different task-specific pipelines to be composed from the same underlying catalog.
- The **MCP interaction layer** exposes the component catalog to the orchestrator through structured tool definitions. Each definition specifies the operation performed by the component, its required inputs and processing state, its configurable parameters, and the execution results or quality control information returned to the orchestrator. The use of structured interfaces reduces ambiguity and enables the orchestrator to invoke established EEG processing functions using explicit parameters without generating MATLAB or EEGLAB implementation code for each operation.
- The **execution environment** performs numerical signal processing as selected by the orchestrator. It maintains the active EEG dataset and executes the corresponding EEGLAB and MATLAB functions. After each processing step, it returns execution results and, where applicable, quality control measures to the orchestrator in order to inform subsequent processing decisions. Following completion of the workflow, the framework produces the processed EEG dataset, derived features where requested, and a provenance record containing the executed operations, parameter values, intermediate quality control results, and subsequent tool invocations made during pipeline execution.

The architecture intentionally separates three responsibilities: the orchestrator performs task interpretation, pipeline synthesis, and quality control-informed adaptation; MCP provides structured access to the EEG processing component catalog and communicates execution results between the orchestrator and processing environment; and EEGLAB and MATLAB perform the numerical signal processing operations. In this way, MCP serves as the structured interaction layer rather than the source of either the pipeline selection logic or the underlying numerical EEG processing algorithms.

### 3.3. EEG Processing Component Catalog

The component catalog is designed to cover the principal operations required to construct preprocessing and analysis workflows for the EEG tasks considered in this study. The catalog includes components for data loading and inspection, signal preparation, artifact handling, epoch construction, feature extraction, quality assessment, and output generation. This catalog contains more than 40 reusable components and is extensible, allowing users to add other utilities, preprocessing tools, and analysis tools as components. There are several classes of components:

**Data management components** provide the orchestrator with the basic utilities for loading BIDS-formatted EEG datasets and individual recordings, inspecting dataset metadata, preserving channel location information, and maintaining the current processing state. These components provide the orchestrator with information such as the number of channels and samples, sampling frequency, recording duration, event structure, and available recording or experimental metadata.

**Signal preparation components** support resampling, high-pass filtering, low-pass filtering, band-pass filtering, line noise removal using CleanLine, and re-referencing. Configurable parameters are exposed through the MCP interface; for example, filtering components require the orchestrator to specify the relevant cutoff frequencies, while the CleanLine component accepts an explicit list of line frequencies to be removed. If no line frequencies are specified, the component defaults to the mains frequency reported in the BIDS metadata when this is available. Additional harmonic frequencies are not added automatically, but could be specified by the orchestrator.

**Artifact processing components** include a configurable artifact cleaning tool based on pop_clean_rawdata and an ICA-based artifact correction tool. The artifact cleaning tool performs bad channel detection by default; when its channels_only option is disabled, ASR burst correction and window rejection are additionally enabled using the specified burst and window criteria. The ICA-based artifact correction tool internally performs ICA decomposition, automatic component classification using ICLabel, removal of components meeting the specified rejection criteria, a post-ICA pop_clean_rawdata pass using configurable burst and window criteria, and interpolation within a single tool invocation.

**Epoch processing components** support segmentation of continuous recordings and rejection or interpolation of contaminated data according to the available processing criteria. For epoched data, a channel is flagged as contaminated if any sample within the epoch exceeds the specified voltage threshold. The proportion of flagged channels is then compared with the predefined epoch_interp_max_chans_pct criterion. If the proportion of contaminated channels exceeds this threshold, the entire epoch is flagged for rejection; otherwise, only the contaminated channels are interpolated within that epoch using spherical interpolation. Epochs flagged for rejection are subsequently removed using pop_rejepoch. This procedure allows spatially localized high-amplitude contamination to be corrected while reserving whole-epoch rejection for epochs with more widespread channel contamination.

**Analysis components** support power spectral density estimation, frequency-band feature extraction, spectral entropy calculation, and generation of task-relevant outputs. Additional components save processed datasets and the associated processing records.

Each component in the catalog is described using five types of information:

1. The operation performed by the component.
2. The input data or processing state required for execution.
3. The configurable parameters exposed to the orchestrator.
4. The output or state modification produced by the component.
5. The processing summary or quality control information returned to the orchestrator.

These structured descriptions enable the orchestrator to reason about component selection, parameterization, compatibility, and execution dependencies. For example, channel location information must be preserved in order to enable interpolation of rejected channels, ICA can only be performed after the required preprocessing state has been established, and spectral features are extracted only after the selected preprocessing operations have been completed. Processing summaries and quality control measures returned by individual components are incorporated into the active processing context. This information can be used by the orchestrator to revise component selection, parameter settings, or execution decisions, including rerunning or reparameterizing previously invoked components when appropriate.

A summary of the principal components is provided in Table 1.

### 3.4. Agentic Pipeline Synthesis Process

Pipeline synthesis begins when the user supplies an EEG analysis task and identifies the dataset to be analyzed. The orchestrator then proceeds through four interrelated stages: task interpretation, dataset inspection, pipeline construction, and iterative pipeline execution with quality control feedback. Although an initial processing plan is constructed before execution, intermediate processing summaries and quality control information returned by individual components can be incorporated into the active processing context and used to revise processing decisions, including rerunning or reparameterizing previously invoked components when the returned results indicate that additional or modified processing is warranted.

#### 3.4.1. Task Interpretation

The orchestrator first interprets the intended analytical outcome. This step identifies the signal characteristics and processing requirements relevant to the downstream task and the processing capabilities likely to be needed. For a resting state spectral analysis task, for example, the orchestrator can identify the need to attenuate line interference and non-neural artifacts while preserving the frequency range relevant to the requested spectral features. The task description does not specify a complete processing sequence, identify individual processing components, or provide parameter values; consequently, the orchestrator is required to translate the requested analytical objective into a set of processing decisions rather than reproducing a user-defined workflow.

#### 3.4.2. Dataset Inspection

The orchestrator next inspects the dataset and available recording metadata. In the present experiment, dataset inspection is performed using the get_eeg_info, get_event_info, and get_eeg_snapshot tools. The get_eeg_info tool returns recording state information, including the number of channels, number of samples, number of trials, sampling frequency, temporal bounds, and whether an ICA decomposition is present. The get_event_info tool returns the available event types and their counts. The get_eeg_snapshot tool returns the dimensions of the EEG data matrix together with a small raw data sample, which is primarily used to verify data integrity and identify issues such as missing or invalid values. By default, the snapshot consists of a small raw data sample (three channels by ten samples), and is used only for basic integrity checks rather than signal characterization.

These inspection steps allow subsequent pipeline decisions to be conditioned on recording metadata, event structure, and basic data integrity rather than being selected solely from a generic task description; for example, the mains frequency reported in the BIDS metadata can inform the frequencies targeted for line noise removal, the sampling frequency constrains valid filter and resampling settings, and the presence and structure of event markers inform how epochs can be constructed.

Additional signal-derived diagnostics, including spectral estimates, amplitude-based summaries, channel quality measures, and line noise characteristics, are available through other processing components in the catalog but are not part of the initial dataset inspection stage in the present experiment. Such measures can be computed later during processing or quality control evaluation when required by the orchestrator.

#### 3.4.3. Component Selection and Composition

Using the task interpretation and dataset information, the orchestrator selects components from C, configures their parameters, and arranges them in an executable order. A valid pipeline has to satisfy the input and processing state requirements of its constituent components, making component ordering an explicit part of the synthesis problem. Where applicable, tool definitions and execution state checks constrained component invocation to compatible processing states. Thus, the orchestrator operates within the processing capabilities and parameter constraints exposed by the catalog rather than generating arbitrary signal processing code.

The resulting plan specifies both the processing operations and their parameter configurations. For example, a plan can specify the target sampling frequency, frequencies passed to CleanLine, filter cutoff frequencies, artifact cleaning strategy, re-referencing scheme, ICA-based artifact correction settings, epoch duration, and spectral or other features to be extracted. The initial plan can subsequently be modified during execution in response to intermediate processing summaries and quality control information.

#### 3.4.4. Execution, Quality Control Feedback, and Provenance Capture

After constructing the initial pipeline, the orchestrator invokes the selected components through the Model Context Protocol (MCP) in the planned order. The execution environment maintains the current EEG dataset and processing state across successive operations, allowing the output of one component to become the input to the next.

Each invocation returns an execution result together with a processing summary and, where applicable, quality control information. These outputs are incorporated into the orchestrator’s active processing context and can inform subsequent processing decisions.

The quality control information available to the orchestrator depends on the invoked component, and includes structured outputs such as channel counts before and after artifact cleaning, number of rejected or interpolated channels, artifact cleaning status, ICA execution status and component removal summaries, epoch retention and rejection counts, and spectral or other analysis outputs when those components are called. During the initial dataset inspection stage, the orchestrator primarily receives recording metadata, event information, and a limited raw data integrity snapshot rather than detailed signal-derived diagnostics.

The orchestrator is permitted to invoke a previously executed tool again, including with revised parameter values, when an execution error occurs or when the returned processing summaries or quality control information suggests that a different parameterization or additional processing step is warranted. Thus, adaptive revision is not restricted only to future operations; previously executed components can also be rerun or reparameterized as part of the same processing workflow.

Rather than applying a single global set of hard-coded quality control thresholds across all tools, the orchestrator interprets the returned processing summaries and quality control information in the context of the requested analysis and the current processing state. Tool-specific criteria and parameter constraints remain defined by the individual processing components, while the orchestrator can use the returned results to determine whether to proceed, revise parameters, repeat an operation, or select an alternative subsequent component.

The framework records the component name, parameter values, execution order, returned status, intermediate quality control results, and subsequent tool invocations. The resulting provenance record provides the operations executed, their parameter configurations, and the processing information returned during workflow execution.

In the present study setup, the orchestrator synthesizes a single pipeline within one interactive session, then applies it to the data. Evaluation is focused on whether the generated workflow can be executed without manual modification of its processing sequence or parameter configuration and whether its output can be compared with that of an established EEG processing pipeline. Systematic analysis of variability across repeated independent synthesis runs was not included in the primary evaluation.

### 3.5. Implementation

The MCP server was implemented in Python 3.10.20 [36] using FastMCP 3.1.1 [37]. Communication with EEGLAB was implemented through MATLAB Engine API for Python 26.1.12 using MATLAB R2026a (26.1.0.3251617, Update 2) [35]. EEG processing was performed using EEGLAB 2026.0.0 [34] together with CleanLine v2.1 [38,39], ICLabel v1.7 [40], and clean_rawdata/ASR v2.11 [41,42]. The implementation was executed on Windows 11 (64-bit) [43].

The persistent session maintains the currently loaded EEG dataset and associated processing state across successive tool invocations. This avoids repeatedly loading the same recording or re-initializing MATLAB during a multistage workflow. It also allows the orchestrator to construct and execute a pipeline incrementally while preserving the effects of earlier operations.

EEGLAB and MATLAB functions are wrapped using structured interfaces that accept named parameters and return standardized execution results. All numerical transformations of the EEG signals are performed by the underlying functions rather than the language model. Errors produced by MATLAB, EEGLAB, or the wrapper layer are returned to the orchestrator in a structured form. In this way, execution errors are available to the orchestrator as part of the active processing context, allowing it to revise parameters or repeat the failed operation when appropriate.

#### Agent and LLM Configuration

The orchestrator was Claude Sonnet 4.6 (Anthropic), accessed through the Claude desktop application, which connected to the EEG processing MCP server as a local tool provider. The model was run using the application’s default configuration: the reasoning effort control was left at its default setting (“low”) and no numerical temperature or top-*p* parameter was exposed to the user, so no sampling parameters were modified. The agentic evaluation was conducted in April 2026.

The orchestrator received a single natural language task prompt instructing it to determine an EEG processing pipeline for resting state recordings and to support extraction of electrophysiological features relevant to the requested analysis, including spectral and network-level measures. The verbatim prompt is reproduced in Appendix B.1. The prompt specified the analysis objective and dataset characteristics, namely, the BIDS organization of the data (with an empty BIDS task label) and the recording location (Germany), which is informative about the local mains frequency. It did not prescribe a processing sequence, name specific tools, or supply processing parameter values. The requested features were chosen to correspond to the outputs of DISCOVER-EEG so that the resulting pipelines could be compared; no other information about DISCOVER-EEG, including its configuration or previously obtained results, was provided to the orchestrator.

Tool definitions were exposed through the Model Context Protocol, and each schema included its parameters together with the default values defined by the underlying function (for example, default ICLabel rejection thresholds, a default Welch window, and a default burst criterion). The orchestrator adopted several of these defaults. The agentic loop was managed by the client application, while the model selected and invoked tools autonomously; execution proceeded until the model determined that the task was complete. Failed tool invocations returned a structured error object to the orchestrator, allowing it to modify or retry the invocation within the same session.

All eight recordings were processed within a single conversation. The pipeline was synthesized once at the start of the session; the agent then executed it per recording, pausing for user confirmation between recordings. Because the full interaction history remained in the model’s context across recordings, the orchestrator could inspect the outputs of earlier steps and earlier recordings when making subsequent decisions; thus, the context was shared and cumulative across recordings.

### 3.6. Experimental Design

The evaluation consisted of three stages. The first experiment assessed the execution fidelity of the MCP-based tool layer by reproducing an established EEG processing workflow through the structured MCP interfaces and comparing the resulting outputs with those obtained from the original implementation. This stage was designed to verify that the tool-mediated execution architecture could reproduce the intended signal processing operations independently of pipeline decisions made by the agent.

The second stage evaluated agentic pipeline synthesis. The orchestrator was provided with a resting state EEG analysis objective, information about the input recordings, and access to the available EEG processing component catalog, but was not provided with the processing sequence or parameter configuration used by the reference pipeline. The orchestrator independently selected, parameterized, and ordered the available components to construct an executable EEG processing workflow. The resulting pipeline was then compared with DISCOVER-EEG using the same input recordings and common downstream evaluation procedures.

A separate downstream evaluation subsequently examined whether EEG processed using the agent-generated pipeline retained information relevant to distinguishing individuals with Alzheimer’s disease from healthy controls. The evaluation addressed the following research questions:

**RQ1—Tool-layer execution fidelity:** Can an established EEG processing workflow executed through the MCP-based tool interface reproduce the outputs obtained from the original implementation of the same processing operations?
**RQ2—Pipeline synthesis:** Can the agentic orchestrator independently select, parameterize, and order available EEG processing components to generate a valid and fully executable pipeline from a user-defined analysis objective and characteristics of the input EEG data?
**RQ3—Pipeline quality:** How does the agent-generated pipeline compare with an established automated pipeline in data retention, artifact attenuation, and preservation of task-relevant spectral features?
**RQ4—Clinical pipeline synthesis:** Can the agentic framework synthesize an executable EEG pipeline that supports downstream clinical reasoning?

#### 3.6.1. Tool-Layer Execution Fidelity Condition

To evaluate the execution fidelity of the MCP-based tool layer, the processing sequence and parameter configuration of the reference DISCOVER-EEG workflow were implemented using the corresponding EEG processing components exposed through the MCP interface. In this condition, the processing operations and their parameters were specified according to the reference workflow rather than being selected by the agent. The purpose of this condition was not to evaluate pipeline synthesis but to determine whether execution of established EEG processing operations through the MCP-based architecture introduced discrepancies relative to their original implementation.

The same input EEG recordings were processed using the original reference implementation and the corresponding MCP-mediated implementation. Where equivalent operations were available, processing parameters were held constant between implementations. Final preprocessed outputs were compared using four families of fidelity measures: (1) recording-level metadata (channel count, samples per epoch, sampling rate, epoch window, and retained epoch/trial count); (2) per-channel descriptive statistics (mean, standard deviation, RMS) and per-channel Pearson correlation between the two implementations’ time series; (3) absolute and percent differences in band power (delta, theta, alpha, beta, gamma); and (4) global point-wise agreement across all channels and samples, summarized as mean absolute error (MAE), root mean square error (RMSE), relative RMSE, and a global Pearson correlation coefficient. Agreement between the two implementations was assessed against a prespecified numerical tolerance; a recording was classified as in close agreement if the global Pearson correlation was ≥0.999 and the relative RMSE was ≤5%.

To ensure that differences between implementations could not arise from stochastic processing steps, both implementations used controlled random initialization. A shared function derived a deterministic random state for each randomized step from a base seed (42), the recording identifier, and the processing step, so that corresponding steps received identical random states in both implementations regardless of parallel execution or processing order. This applied to RANSAC-based bad channel detection. ICA decompositions were deterministic in both implementations because, in the EEGLAB version used and with the settings applied, runica resets its random number generator to a fixed state at each call. Both implementations also executed ICA single-threaded and performed average re-referencing without re-adding the reference channel, matching the reference configuration.

This evaluation provides a control for the subsequent agentic pipeline comparison by distinguishing differences attributable to the MCP-based execution layer from differences arising from the processing operations and parameter values independently selected by the agent.

#### 3.6.2. Agentic Pipeline Synthesis Condition

The orchestrator was provided with three inputs: a natural language description of a resting state EEG analysis task, access to the BIDS-formatted EEG recordings and their metadata, and access to the catalog of available EEG processing components. The task description specified the intended analysis but did not prescribe a processing sequence, identify particular components, or provide parameter values; therefore, this condition evaluates RQ2 by requiring the orchestrator to construct the processing sequence and parameter configuration independently from the available task description, dataset information, and component catalog.

The orchestrator was instructed to inspect the dataset and generate a complete EEG processing pipeline. It was responsible for selecting the processing components, configuring their parameters, and determining their execution order. The generated pipeline was subsequently executed through the MCP-based component interface.

Information about DISCOVER-EEG, including its processing sequence, parameter configuration, implementation details, and previously obtained results, was excluded from the orchestrator’s context. This separation prevented direct access to the reference implementation or its study-specific configuration during pipeline synthesis.

For RQ2, a generated pipeline was considered valid and executable if it met the following conditions:

1. Contained the operations required to transform the input recordings into the requested processed outputs.
2. Used components and parameters compatible with the input data.
3. Satisfied the execution dependencies among the selected components.
4. Executed from beginning to end without manual modification while permitting agent-directed tool retries or reparameterization based on execution results or quality control feedback.
5. Produced a complete processed dataset and pipeline record.

#### 3.6.3. Reference Condition

DISCOVER-EEG was selected as the reference condition because it provides an established automated workflow for preprocessing and analyzing resting state EEG recordings. The orchestrator was not informed that its generated pipeline would be compared with DISCOVER-EEG.

Each input recording was processed separately using the agent-generated pipeline and DISCOVER-EEG. Both pipelines received the same original recordings, and their outputs were evaluated using the same downstream procedures. All comparative metrics were computed using the same analysis code and common channel set for both pipeline outputs. Because the two pipelines could contain different processing operations and parameter configurations, their output signals were not expected to be numerically identical.

For RQ3, the comparison examined three dimensions of pipeline quality:

1. **Data retention**, measured by the proportion of candidate epochs retained after preprocessing.
2. **Artifact attenuation**, assessed using overall and band-specific signal-to-noise measures.
3. **Spectral preservation**, assessed using spectral entropy and the distribution of power within the task-relevant EEG frequency range.

Results were evaluated across all recordings and separately inspected for the eyes-open and eyes-closed conditions.

### 3.7. Resting State EEG Dataset

The evaluation used resting state EEG data from four participants recorded under eyes-open and eyes-closed conditions, resulting in eight recordings. The data were the publicly available demonstration subset of the chronic pain resting state EEG dataset of Zebhauser, Gil Ávila, and Ploner [44], organized according to the Brain Imaging Data Structure (BIDS, version 1.9.9) and distributed under a CC BY-NC-SA license. The participants were four patients with chronic pain (one male, three female, ages 32–67 years, recorded in Munich, Germany). In each condition, participants were instructed to remain awake and relaxed without performing any particular task.

The recordings were acquired with a CGX Quick-32r mobile dry-electrode system and BrainVision Recorder for CGX software (v. 2.00.0010; Brain Products, Munich, Germany), using 29 EEG channels positioned according to the 10–20 system (Fp1, Fpz, Fp2, AF7, AF8, F7, F3, Fz, F4, F8, FC5, FC6, T7, C3, Cz, C4, T8, CP5, CP6, P7, P3, Pz, P4, P8, PO7, PO8, O1, Oz, O2), referenced to and grounded at A1 (left ear). Signals were digitized at 500 Hz with no online filtering, and the local mains (line noise) frequency was 50 Hz. Each recording lasted approximately five minutes. Data were stored in BrainVision format (.vhdr/.vmrk/.eeg).

The same eight recordings were used in the agentic pipeline synthesis and reference conditions, permitting paired comparisons at the recording level. All downstream comparisons were performed over the same 29 EEG channels, meaning that the spatial channel set was identical between pipeline outputs.

### 3.8. Evaluation Metrics

Candidate epochs were generated using segments of 2 s with 50% overlap. Identical epoch boundaries were used for both pipeline outputs, and $N_{\mathrm{candidate}}$ was defined before pipeline-specific rejection so that differences in retention reflected rejection rather than differences in epoch construction.

Epoch retention was calculated as

(3) $$R_{\mathrm{epoch}}=\frac{N_{\mathrm{retained}}}{N_{\mathrm{candidate}}},$$

where $N_{\mathrm{retained}}$ is the number of epochs remaining after preprocessing and $N_{\mathrm{candidate}}$ is the number of candidate epochs available before epoch rejection.

Power spectral density (PSD) was estimated separately for each channel and 2 s epoch using MATLAB’s pwelch function with a single Hanning window spanning the full epoch. At the 250 Hz analysis sampling rate, each epoch comprised 500 samples; the FFT length was left at the pwelch default, which for an empty specification is the greater of 256 or the next power of two at least as large as the window length, giving 512 DFT points and a frequency resolution of approximately 0.49 Hz. PSD estimates were first averaged across epochs within each channel to obtain one spectrum per channel, and were subsequently averaged across the common channels to obtain a single recording-level mean PSD for each pipeline.

Spectral entropy was calculated over the 1–30 Hz frequency range. The 1–30 Hz range was used for the resting state comparison with DISCOVER-EEG, and was retained consistently across both pipeline outputs for this evaluation. Spectral entropy was calculated from the channel-averaged recording-level PSD rather than separately at the channel level. The PSD within 1–30 Hz was normalized to sum to one before calculation of the Shannon entropy.

Let $S_{i}$ denote the spectral power at frequency bin *i*, and let

(4) $$p_{i}=\frac{S_{i}}{\sum_{j=1}^{M}S_{j}}$$

denote the normalized spectral power. Spectral entropy was then calculated as

(5) $$H=-\sum_{i=1}^{M}p_{i}\log_{2}\left(p_{i}\right).$$

This measure was used to quantify differences between the two pipelines in the distribution of spectral power within the frequency range relevant to the resting state analysis.

The same PSD estimates were used for calculation of frequency-band power, spectral entropy, and SNR-based measures to ensure that all spectral comparisons were derived from a common spectral estimation procedure.

The overall signal-to-noise ratio was calculated as

(6) $$\mathrm{SNR}=10\log_{10}\left(\frac{P_{\mathrm{signal}}}{P_{\mathrm{noise}}}\right),$$

where $P_{\mathrm{signal}}$ and $P_{\mathrm{noise}}$ denote the mean power spectral density (in $\mu V^{2}/\mathrm{Hz}$) averaged across frequency bins within the 1–30 Hz signal band and the 40–60 Hz noise band, respectively, then averaged across channels. Band-specific SNR values for the delta (1–4 Hz), theta (4–8 Hz), alpha (8–13 Hz), and beta (13–30 Hz) bands were computed with the same expression, substituting the corresponding frequency range for the signal band while retaining the 40–60 Hz noise band.

Because the agent-generated and reference pipelines could use different filter boundaries, SNR was interpreted together with epoch retention and spectral entropy; this prevents conclusions about pipeline quality from relying exclusively on attenuation within the designated noise frequency range.

### 3.9. Statistical Analysis

Statistical analyses were conducted at the recording level because each EEG recording was processed using both the agent-generated and reference pipelines, yielding paired measurements for each processing outcome. For each metric, recording-level values and within-recording differences between pipelines were retained to show the consistency and magnitude of the observed effects across individual recordings.

Given the small number of recordings and the exploratory proof-of-concept nature of the evaluation, statistical interpretation emphasized descriptive statistics, paired differences, effect sizes, and confidence intervals rather than relying solely on null hypothesis significance testing. For each pipeline, results are reported as the mean and standard deviation together with the mean paired difference and its 95% confidence interval. Cohen’s $d_{z}$ was calculated as the mean paired difference divided by the standard deviation of the paired differences. Recording-level paired observations were also visualized to allow direct assessment of between-recording variability and the consistency of pipeline effects.

Paired-samples *t*-tests were used as exploratory inferential comparisons of the agent-generated and reference pipelines. The tests were applied to the within-recording differences rather than to the raw measurements from each pipeline. Because of the limited sample size, formal normality testing was not used as a decision rule for selecting the statistical test; instead, the distribution of paired differences was inspected descriptively for extreme asymmetry or influential observations. Wilcoxon signed-rank tests were additionally performed as sensitivity analyses to determine whether the direction of the findings depended on the parametric test assumption.

Statistical tests were two-sided, with p<0.05 used as the nominal significance threshold. Effect sizes and 95% confidence intervals are reported alongside *p*-values. Because the eight recordings were obtained from four participants under eyes-open and eyes-closed conditions, the recording-level observations are not statistically independent at the participant level; accordingly, the paired inferential analyses should be regarded as exploratory and descriptive of the evaluated recordings rather than as confirmatory population-level inference. Results are interpreted primarily in terms of effect magnitude, confidence intervals, consistency across individual recordings, and agreement between the parametric and nonparametric sensitivity analyses. In addition, because all recordings were processed within a single interactive session using one synthesized pipeline, the recording-level outcomes reflect repeated application of the same agent-generated pipeline rather than independent pipeline syntheses; run-to-run variability of the synthesis process itself was not assessed.

### 3.10. Clinical Pipeline Synthesis for Downstream Clinical Reasoning Tasks

We conducted a follow-up experiment to assess whether the agentic framework could synthesize a pipeline that supports a downstream clinical reasoning task of distinguishing individuals with Alzheimer’s disease from healthy controls using EEG-derived biomarkers. This required the orchestrator to interpret a clinical objective, identify a suitable set of biomarkers, and synthesize an EEG processing pipeline that retained the information needed for the clinical assessment.

Each participant in this experiment was analyzed in a separate conversation with no shared context. In each conversation, the orchestrator was presented with the participant’s eyes-closed and eyes-open recordings as being those of a patient undergoing cognitive assessment. It was instructed to discover the available tools, determine and execute an appropriate preprocessing and analysis workflow, evaluate data quality, extract relevant EEG biomarkers, compare the eyes-closed and eyes-open findings, and provide a clinical interpretation justified by the computed biomarkers. The prompt also provided acquisition details, including the recording reference and the photic stimulation protocol of the eyes-open recordings. The prompt is reproduced in Appendix B.2; it retains the original instructions, but generalizes file paths and dataset-specific identifiers. The orchestrator was not provided with the candidate diagnostic categories. Impressions indicating Alzheimer’s-type or neurodegenerative changes were classified as AD, while impressions indicating a normal EEG or no significant abnormality were classified as HC.

Classification was performed in a zero-shot setting. The orchestrator reached its clinical impression from the EEG-derived features extracted by the synthesized pipeline, its own domain knowledge, and retrieved EEG literature [45,46,47,48,49,50,51,52,53,54,55,56,57,58,59,60,61,62,63,64,65,66]. No labeled training data, fine-tuning, classifier training, threshold optimization, or feature selection was performed, and diagnostic labels were used only to evaluate the resulting predictions. This experiment was exploratory and its purpose was to assess whether an agent-synthesized workflow could support an end-to-end clinical reasoning task; as such, it was not intended to maximize classification performance.

#### Alzheimer’s Disease Dataset

The Alzheimer’s disease datasets used for this study, commonly referred to as ds004504 and ds006036 on OpenNeuro, were acquired by neurologists from the Second Department of Neurology of AHEPA General Hospital of Thessaloniki [67,68]. Of the participant population, 36 were diagnosed with Alzheimer’s disease (AD), 23 were diagnosed with frontotemporal dementia (FTD), and 29 were healthy subjects. For the purposes of this paper, the FTD group was omitted. Within the AD group, the duration of the disease was measured in months with the median time being 25. The mean age of the AD group was 66.4 years, with a standard deviation of 7.9 years; for the control group, the mean age was 67.9 years with a standard deviation of 5.4 years.

Recordings for the ds004504 dataset were taken on a Nihon Kohden EEG 2100 clinical device, including 19 scalp electrodes (Fp1, Fp2, F7, F3, Fz, F4, F8, T3, C3, Cz, C4, T4, T5, P3, Pz, P4, T6, O1, and O2) according to the 10–20 international system, with two reference electrodes (A1 and A2) placed on the mastoids. Each recording was taken in accordance with clinical guidelines, with patients seated and their eyes closed. Before each recording, we ensured that the skin impedance value was below 5 kΩ. The sampling rate of the system was 500 Hz; amplifier sensitivity was set to 10 μV/mm resolution. Recording montages used anterior–posterior bipolar and referential montage, using Cz as the common reference. Recordings were taken under the range of the following parameters of the amplifier: sensitivity 10 μV/mm, time constant 0.3 s, and high frequency filter at 70 Hz. For the AD group, each recording lasted approximately 13.5 min, with a minimum of 5.1 min and a maximum of 21.3 min. The control group had a mean of 13.8 min per recording, with a minimum of 12.5 min and a maximum of 16.5 min. A total of 485.5 min of AD recordings were taken, and a total of 402 min of recordings were available for the control group.

Meanwhile, the ds006036 dataset served as a complement to the ds004504 dataset; the same subjects and device were used. For the purposes of this study, the FTD group was again omitted. This time, participants were seated with their eyes open while being exposed to photic stimulation. This was administered at incremental frequencies, beginning from 5 Hz to 10 Hz, then to 15 Hz and in some cases up to 30 Hz. Each recording lasted approximately 4.86 min for the AD group, while for the control group the recordings lasted 6.43 min on average. This totaled to 174.94 min of recordings included in the dataset for the AD group and 186.50 min for the control group.

## 4. Results

### 4.1. RQ1: Tool-Layer Execution Fidelity

With controlled random initialization, the MCP-mediated and original DISCOVER-EEG implementations produced identical outputs for all eight resting state recordings. For every recording, both pipelines returned the same 29 channels, a sampling frequency of 250 Hz, 500 samples per epoch, an epoch interval of 0–1.996 s, and the same number of retained epochs. Retained epoch counts ranged from 148 to 301 across recordings, with no within-recording difference between pipelines. No channels were present exclusively in either output.

Channel-level descriptive statistics were also identical. Across the two implementations, channel means, standard deviations, and root mean square (RMS) amplitudes did not differ between implementations, and band power in the delta, theta, alpha, beta, and gamma bands showed no difference in any recording.

Pointwise agreement was consistent across all recordings. Every recording yielded a mean channel correlation and global correlation of r=1.0000, a mean absolute error (MAE) and root mean square error (RMSE) of 0μV, and a relative RMSE of 0%, satisfying the prespecified criterion for close agreement (global r≥0.999, relative RMSE ≤5%) in all eight recordings. The outputs were identical sample per sample, with a maximum absolute difference of 0μV. Table 2 summarizes the recording-level comparisons.

Overall, under controlled random initialization, the MCP-mediated workflow reproduced the outputs of DISCOVER-EEG exactly at the structural, channel, spectral, and pointwise levels. These results support the execution fidelity of the MCP tool layer when given the same data, parameters, and random seed.

### 4.2. RQ2: Agentic Pipeline Synthesis

The orchestrator generated a complete EEG processing pipeline without direct access to the DISCOVER-EEG processing sequence, study-specific parameter configuration, or previously obtained outputs. The synthesized pipeline first loaded the BIDS-formatted recording, preserved the original channel locations, and resampled the EEG signals to 250 Hz. It then performed line noise removal and frequency filtering, followed by artifact cleaning and common-average re-referencing. Subsequently, the pipeline applied ICA-based artifact correction, including ICA decomposition, ICLabel-based component classification, artifact component removal, post-ICA artifact cleaning, and interpolation within the combined ICA processing component. The workflow then segmented the continuous recording into epochs, applied epoch-level artifact handling, and saved the processed dataset and associated pipeline record.

The generated pipeline completed processing for all eight recordings without manual modification of the processing sequence or parameter values. Its component ordering satisfied the required processing dependencies: channel location information was preserved before operations requiring later interpolation, filtering and continuous-data artifact handling preceded ICA-based artifact correction, and epoch construction and rejection were performed after continuous-data preprocessing. These results demonstrate that the orchestrator produced an executable and dependency-consistent workflow rather than an unordered collection of processing operations. While user confirmation was required in order to initiate processing of each subsequent recording, no processing decisions or parameter values were manually changed between recordings.

Although the architecture permits previously executed components to be rerun or reparameterized in response to returned quality control information, this capability was only exercised to a limited extent. Re-invocations of tools during the resting state run occurred in response to execution errors such as tool timeouts and unavailable files. Returned quality control information, including channel counts after artifact cleaning, ICA completion status, and epoch retention, primarily confirmed successful processing, and no processing parameter was revised in response to a data quality measure.

As shown in Table 3, the orchestrator made two notable parameterization decisions that were not specified in the task description. First, it selected 50 Hz and 100 Hz for CleanLine removal. The 50 Hz fundamental was consistent with the mains frequency reported in the BIDS metadata, whereas 100 Hz was added by the orchestrator as the first harmonic. Second, the orchestrator selected a 1–45 Hz band-pass filter before ICA-based artifact correction.

These choices differed from the DISCOVER-EEG reference workflow, which targeted the 50 Hz fundamental but did not include the same 100 Hz CleanLine operation or the same 45 Hz upper filter boundary.

These findings answer RQ2 affirmatively for the evaluated setting: the orchestrator independently selected, parameterized, and ordered the available EEG-processing components, then generated a workflow that executed successfully across all eight resting state recordings without manual intervention.

### 4.3. RQ3: Pipeline Quality

#### 4.3.1. Data Retention and Spectral Preservation

The agent-generated pipeline retained a mean proportion of 0.845 of the available epochs, compared with 0.843 for DISCOVER-EEG. The mean within-recording difference was 0.002. The observed difference in epoch retention was small and not statistically detectable under the exploratory paired comparison (p=0.891).

Mean spectral entropy over the 1–30 Hz range was 4.867 bits for the agent-generated pipeline and 4.841 bits for DISCOVER-EEG, corresponding to a mean difference of 0.026 bits. This difference was also not statistically detectable under the exploratory paired comparison (p=0.236).

As shown in Table 4, the two pipelines showed small mean differences in epoch retention and spectral entropy across the evaluated recordings. These findings provide context for the SNR analyses reported below: although the pipelines differed in their filter boundaries and artifact processing decisions, the higher SNR values observed for the agent-generated pipeline were not accompanied by substantially greater data rejection or large differences in spectral entropy.

#### 4.3.2. Overall Signal-to-Noise Ratio

Under the SNR formulation defined in the Methods section, the agent-generated pipeline achieved a mean overall SNR of 13.92 dB, compared with 11.14 dB for DISCOVER-EEG. This corresponded to a mean difference of 2.78 dB in favor of the agent-generated pipeline. The paired comparison yielded t(7)=16.15, p<0.001, with a 95% confidence interval for the mean difference of [+2.37,+3.19] dB and a large effect size ($d_{z}=5.71$).

The agent-generated pipeline produced higher overall SNR values across the evaluated recordings, as mentioned in Table 5, and the same direction of difference was observed under both the eyes-open and eyes-closed conditions. This pattern indicates that the observed aggregate difference was not confined to a single recording condition.

The agent-generated pipeline included a 45 Hz low-pass boundary, whereas DISCOVER-EEG did not impose the same upper cutoff. Because this filtering decision attenuated part of the frequency range contributing to the noise estimate, the observed SNR difference reflects the complete outputs of the two pipelines, and should not be interpreted as isolating the effectiveness of any single artifact-removal operation. The SNR findings were interpreted together with epoch retention and spectral entropy.

#### 4.3.3. Band-Specific Signal Quality

The agent-generated pipeline also produced higher SNR values within each of the four classical EEG frequency bands evaluated. The mean paired differences were 2.71 dB for delta, 2.72 dB for theta, 2.85 dB for alpha, and 2.99 dB for beta, each with p<0.001. The direction of the difference was consistent across all four bands.

The direction of the SNR difference was consistent across the evaluated frequency bands and was observed under both recording conditions. Under the study’s specified SNR formulation, the observed difference was not confined to a single portion of the 1–30 Hz signal range as demonstrated in Table 6. As with the overall SNR analysis, these results should be interpreted in the context of the different filter boundaries used by the two pipelines.

### 4.4. EEG Pipeline Synthesis for Downstream Clinical Reasoning

The final experiment examined whether the agentic approach could synthesize and execute an EEG processing workflow for a downstream clinically relevant analysis. Specifically, the evaluation assessed whether EEG processed using the agent-generated pipeline retained information relevant to distinguishing participants with Alzheimer’s disease (AD) from healthy controls (HC).

For this analysis, the agentic approach synthesized a pipeline that extracted spectral features using the calculate_band_power component. Power spectral density (PSD) was estimated separately for each channel and epoch using Welch’s method with a Hanning window. PSD estimates were averaged across epochs within each channel and subsequently across channels to obtain a single mean spectrum for each recording. The implementation used a fixed 1–45 Hz analysis range. This spectral analysis configuration was specific to the downstream biomarker analysis, and differed from the 1–30 Hz range used in the earlier DISCOVER-EEG pipeline comparison.

For spectral entropy, the channel-averaged PSD within 1–45 Hz was normalized to sum to one, yielding the spectral probability distribution $p_{i}$. Shannon spectral entropy was then calculated as

(7) $$H=-\sum_{i=1}^{M}p_{i}\log_{2}\left(p_{i}\right).$$

Within the clinical analyses, adaptation during execution was primarily operational. Re-invocations of tools mainly followed execution errors such as tool timeouts or unavailable files, after which the orchestrator inspected the current processing state before continuing. Returned quality control information primarily confirmed successful processing, and no preprocessing parameter was revised in response to a data quality measure.

The EEG-derived features generated by this pipeline were subsequently used for the classification analysis involving Alzheimer’s disease versus healthy controls. Classification performance was evaluated using the resulting class predictions, with AD treated as the positive class. The orchestrator correctly identified 24 of 36 participants with AD and 26 of 29 HC participants (Table 7). It misclassified 12 participants with AD as HC (false negatives) and three HC participants as AD (false positives). This corresponded to an overall accuracy of 76.9% (50/65), a precision of 0.889, a recall (sensitivity) of 0.667, a specificity of 0.897, and an F1 score of 0.762.

The error profile was asymmetric (Table 8). Only 3 of 29 healthy controls were misclassified as AD, resulting in high precision and specificity; in contrast, 12 of 36 participants with AD were misclassified as healthy controls, yielding a lower sensitivity of 66.7%. Thus, positive AD predictions were relatively reliable (88.9% precision), whereas negative predictions were less effective for ruling out AD.

The feature vector supplied to the classification step consisted entirely of channel-averaged spectral measures; no channel-specific or electrode-level features were included. This reflects the implementation of calculate_band_power, which averages Welch PSD estimates first across epochs within each channel and then across channels before downstream features are derived. For each recording, absolute band power (in $\mu V^{2}$, computed by trapezoidal integration of the PSD) and relative band power (expressed as a percentage of total 1–45 Hz power) were calculated for the delta (1–4 Hz), theta (4–8 Hz), alpha (8–13 Hz), beta (13–30 Hz), and gamma (30–45 Hz) bands. The feature set also included the individual alpha frequency (IAF), defined as the frequency of maximum PSD within 7–13 Hz, and the Shannon spectral entropy defined in Equation (7).

To capture spectral slowing patterns commonly associated with Alzheimer’s disease, six additional features were derived from the absolute band powers: the theta/alpha, delta/alpha, alpha/theta, alpha/beta, and theta/beta ratios, together with the global slowing index (GSI):

(8) $$\mathrm{GSI}=\frac{\delta +\theta }{\alpha +\beta }.$$

The alpha reactivity index (ARI) was additionally calculated from the paired eyes-closed (EC) and eyes-open (EO) absolute alpha power as

(9) $$\mathrm{ARI}=\frac{\alpha_{\mathrm{EC}}-\alpha_{\mathrm{EO}}}{\alpha_{\mathrm{EC}}}.$$

In total, classification was based on a 20-dimensional feature vector per participant: five absolute band powers, total absolute power, five relative band powers, IAF, spectral entropy, five band-power ratios, the GSI, and the cross-condition ARI (Table 9). All features were channel-averaged scalar measures; no spatial or topographic information was retained in the classification input.

These findings provide an initial indication that the agent-generated pipeline preserved EEG-derived information relevant to the downstream biomarker task. Given the sample size and exploratory nature of the analysis, the results should be interpreted as a feasibility demonstration rather than as evidence of clinical diagnostic performance.

### 4.5. Summary of Results

Across the evaluated experiments, the MCP-mediated implementation exactly reproduced the outputs of the reference workflow under controlled random initialization. The orchestrator independently synthesized and executed a task-specific EEG processing workflow across all eight resting state recordings, without any manual modification of the processing sequence or parameter configuration. Relative to DISCOVER-EEG, the generated workflow showed small mean differences in epoch retention and spectral entropy along with higher overall and band-specific SNR under the study’s specified evaluation procedure.

In the downstream Alzheimer’s disease versus healthy controls analysis, the agent-generated pipeline supported the extraction of EEG-derived spectral features for classification, yielding an accuracy of 76.9%, precision of 88.9%, sensitivity of 66.7%, specificity of 89.7%, and F1 score of 76.2%.

## 5. Discussion

This study evaluated whether an LLM-based orchestrator could synthesize and execute task-specific EEG processing workflows using structured access to established processing components. Across the evaluated experiments, the framework demonstrated tool-layer execution fidelity, independent pipeline synthesis, quantitative comparison with an established automated workflow, and application to a downstream Alzheimer’s disease versus healthy controls biomarker analysis. Together, these experiments provide an initial assessment of both the technical feasibility and scientific utility of agentic EEG pipeline synthesis.

### 5.1. Agentic EEG Pipeline Synthesis

A central finding is that the orchestrator translated a broad analytical objective into a dependency-consistent and executable EEG processing workflow without being provided with a predefined processing sequence or parameter configuration. This supports the feasibility of using an LLM for pipeline-level decision making while constraining numerical execution to the operations available through the structured component catalog.

The task prompt did not identify individual tools or prescribe parameter values, requiring the orchestrator to determine the operations needed to satisfy the analytical objective. Because execution was restricted to explicitly defined components with specified inputs, parameters, and processing state requirements, the framework separates agentic workflow construction from the implementation of the underlying numerical algorithms.

Two parameter choices illustrate this behavior: the orchestrator selected both 50 and 100 Hz for CleanLine removal, with the 50-Hz component corresponding to the mains frequency represented in the dataset metadata and 100 Hz added as the first harmonic; it also selected a 1–45 Hz band-pass filter before ICA-based artifact correction. These choices were not prescribed by the task prompt and differed from the DISCOVER-EEG reference workflow, which did not use the same 100 Hz CleanLine operation or the same 45 Hz upper filtering boundary. The differences demonstrate that the orchestrator did not simply reproduce the reference workflow; in addition, they highlight an important property of task-directed pipeline generation, namely, that reasonable processing choices may differ across workflows even when the downstream analytical objective is similar.

The framework also supports iterative execution rather than requiring the initially generated plan to remain immutable. Processing summaries, quality control information, and structured execution errors are returned to the orchestrator, and previously invoked components can be rerun or reparameterized. However, this capability was exercised mainly for recovery from execution errors, and returned quality control information primarily confirmed successful processing rather than prompting changes to processing parameters. Thus, while the architecture supports QC-driven adaptation by design, such adaptation was not systematically demonstrated in the present study, and it remains to be established how often it occurs and whether it improves processing outcomes relative to a non-adaptive strategy.

### 5.2. Comparison with DISCOVER-EEG

The comparison with DISCOVER-EEG showed that the independently synthesized workflow achieved small descriptive differences in epoch retention and spectral entropy in the evaluated recordings while producing higher SNR values under the specified metric. Importantly, the small differences in retention and spectral entropy suggest that the higher SNR was not accompanied by substantially greater data rejection or a large alteration in overall spectral complexity.

The SNR results require more careful interpretation. Under this study’s specified SNR formulation, the agent-generated pipeline produced a higher overall SNR than DISCOVER-EEG, and the same direction of difference was observed across the evaluated frequency bands and recording conditions. However, this difference cannot be interpreted as evidence that the agent-generated artifact removal procedure was intrinsically superior, as the generated workflow imposed a 45 Hz low-pass boundary, whereas the reference workflow did not use the same upper cutoff. Because the noise estimate included power in the 40–60 Hz range, attenuation introduced by the 45 Hz low-pass filter could directly increase the calculated SNR even in the absence of improved removal of non-neural artifacts. Therefor, the SNR comparison reflects differences between the complete output signals of the two pipelines rather than the isolated effectiveness of any single cleaning operation.

For this reason, epoch retention and spectral entropy provide important complementary information. A pipeline could obtain an apparently favorable noise metric simply by rejecting substantially more data or attenuating a large portion of the measured spectrum. In the present comparison, the small mean differences in epoch retention and spectral entropy provide some evidence against such an extreme tradeoff, although they do not establish equivalence between the two pipelines. Because no equivalence or non-inferiority testing was performed, non-significant paired comparisons should not be interpreted as evidence that the two pipelines produce equivalent or interchangeable EEG outputs.

### 5.3. Tool-Layer Execution Fidelity

Under controlled random initialization, the MCP-mediated implementation reproduced DISCOVER-EEG exactly across all eight recordings at the structural, channel, spectral, and pointwise levels. This indicates that the MCP-based execution layer did not introduce implementation-level discrepancies and that the observed differences between the agent-generated and reference pipelines in RQ3 can be attributed to the processing decisions made by the orchestrator rather than to the tool interface. These results also highlight that exact reproducibility of EEG preprocessing requires controlling stochastic steps and matching execution settings across implementations.

### 5.4. Relationship to Existing Automated and LLM-Assisted EEG Frameworks

Existing automated EEG pipelines such as PREP [12], HAPPE [13], Automagic [14], and DISCOVER-EEG [15] have demonstrated the value of standardized preprocessing and quality control procedures for improving reproducibility and reducing manual processing burden. The principal contribution of the present approach is not the introduction of new EEG algorithms [26,27,28,29,30] but the use of an agentic orchestrator to compose EEG processing operations according to the requested task and available data information, with returned processing state available to inform revisions during execution. The underlying computations remain delegated to existing EEG processing implementations. This design preserves the parameterization and execution provenance while allowing the sequence of operations to vary across analytical objectives.

Recent LLM-based EEG systems have explored related approaches to tool-mediated analysis. BrainAgent [23] uses a hierarchical multi-agent architecture for automated brain signal analysis, NeuraDock [24] separates deterministic local EEG processing from a language model interface, and EEGAgent [25] combines LLM reasoning with EEG analysis tools. In contrast, the present study focuses specifically on the synthesis of an explicit and executable EEG processing pipeline from a catalog of composable operations. The resulting workflow can be inspected as a sequence of parameterized processing steps rather than existing only as a natural language interpretation or final prediction.

This distinction is particularly relevant for neuroscience applications, where the validity of a downstream measure depends on the transformations applied to the acquired signal. Explicit tool invocation makes it possible to inspect which operations were selected, which parameters were used, and what intermediate processing information was returned. Such provenance is important when an LLM-based system participates in scientific signal processing, as the rationale produced by a language model alone is insufficient to establish reproducibility.

### 5.5. Downstream EEG Biomarker Analysis

The downstream analysis of Alzheimer’s disease classification versus healthy controls extends the evaluation beyond direct comparison with a predefined reference workflow by asking whether an agent-generated pipeline can support a clinically oriented biomarker task. The resulting classification performance provides an initial demonstration of end-to-end feasibility, from an analytical objective through EEG processing and feature extraction to a downstream classification result.

The error profile was asymmetric: precision and specificity were relatively high whereas sensitivity was lower, indicating that the approach was more reliable when identifying positive AD classifications than when ruling out AD. This distinction is important because an apparently favorable overall accuracy can obscure clinically relevant differences between false positive and false negative behavior.

Previously published benchmarks on the same ds004504 dataset reported accuracy results of 71.2–77.0% and AD sensitivities of 69.7–78.3% across five classifiers when evaluated with leave-one-subject-out validation [67]. Another study reported an accuracy of 77.6% using a random forest classifier [69]. The zero-shot accuracy obtained here (76.9%) is comparable to these baselines, although these benchmarks were trained on labeled data from the same cohort, used different feature sets, and were evaluated at the epoch level rather than per participant, making the comparison only approximate. The lower sensitivity (66.7%) indicates that missed AD cases remain a limitation of the present approach.

These findings indicate that the synthesized workflow retained EEG-derived information relevant to Alzheimer’s disease versus healthy controls discrimination, although they do not establish diagnostic validity or superiority to conventional preprocessing because the same zero-shot classification was not repeated on EEG processed with a reference pipeline. The analysis should be interpreted as a feasibility demonstration of agent-directed EEG processing and downstream biomarker analysis rather than as evidence of autonomous clinical diagnosis.

### 5.6. Limitations

Several limitations should be considered when interpreting the present results. First, the primary resting state comparison used only eight recordings obtained from four participants. Eyes-open and eyes-closed recordings from the same participant are correlated observations, and should not be interpreted as eight statistically independent participants. The paired statistical tests were consequently treated as exploratory, with emphasis placed on effect sizes, confidence intervals, individual recording-level behavior, and agreement with nonparametric sensitivity analyses rather than on *p*-values alone. The small demonstration sample is appropriate for an initial systems evaluation, but does not support broad conclusions about generalization across participants, acquisition systems, laboratories, or EEG populations.

Second, the orchestrator synthesized only one pipeline within a single interactive session. That pipeline was then applied sequentially to all eight recordings, with user confirmation between recordings. The full interaction history remained available across the session, allowing outputs from earlier recordings to remain in context during later processing. As such, the present study evaluates repeated application of one synthesized pipeline rather than variability across independent synthesis runs; it remains unknown whether repeated prompts issued in fresh contexts would produce the same component sequence, parameter settings, or processing order.

Third, the use of a commercial LLM introduces model- and client-dependent behavior. The orchestrator used Claude Sonnet 4.6 through the Claude desktop application with the application’s default reasoning configuration, and the client controlled the agentic execution loop and stopping behavior. Even with a fixed prompt and tool catalog, future model updates or changes in the client environment could alter tool selection behavior. Reproducibility should include the model identity, client environment, prompt, tool schemas, software versions, and experiment date in addition to the numerical EEG code.

Fourth, the quality control adaptation supported by the framework is primarily LLM-mediated rather than governed by a single global set of numerical thresholds. Tool-specific criteria remain defined by the individual components, but the orchestrator can interpret structured outputs and decide whether to continue, repeat, or reparameterize an operation. While this design provides flexibility, it also introduces a source of variability that was not isolated experimentally. Moreover, a distinction should be drawn between the architecture’s support for QC-driven adaptation and the adaptation that was actually observed. Because the evaluated recordings were largely clean, quality control checks rarely indicated a need to change processing, and adaptation in the reported experiments consisted mainly of recovery from execution errors. No processing parameter was revised in response to a data quality measure. Thus, although the architecture supports QC-driven adaptation, such adaptation was not systematically tested or demonstrated in this study, and the present findings should be regarded only as a preliminary evaluation of this capability.

Fifth, the SNR measure is sensitive to differences in filtering, particularly because the generated pipeline used a 45 Hz low-pass boundary while the designated noise range extended to 60 Hz. Thus, the higher SNR observed for the generated pipeline cannot be interpreted independently of its frequency response characteristics.

Sixth, the pipeline generation space was limited to the tools exposed through the MCP catalog. Although the full catalog includes preprocessing, spectral, connectivity, complexity, source reconstruction, and quality control operations, the orchestrator cannot select an operation that has not been implemented and exposed as a tool. Thus, the framework’s adaptability depends partly on the breadth, correctness, and documentation of the available component library.

Seventh, the Alzheimer’s disease versus healthy controls analysis was a single exploratory zero-shot evaluation, without training, fine-tuning, or optimization for sensitivity. The relatively low AD sensitivity (66.7%) is an important limitation of this evaluation, and indicates that the approach missed a substantial proportion of participants with AD. One-third of participants with AD were classified as healthy controls, and the features that contributed to correct and incorrect classifications were not analyzed. Because the classifications arise from the orchestrator’s reasoning rather than from a trained model, standard feature attribution methods such as SHAP could be applied directly. Identifying which features support or hinder the detection of AD remains an open question.

Finally, the current evaluation focuses primarily on resting state EEG and an exploratory downstream clinical biomarker application. Additional work is needed to determine whether the same pipeline synthesis approach generalizes to event-related potentials, motor imagery BCIs, connectivity analyses, multimodal recordings, developmental EEG, and other paradigms with different signal preservation requirements.

### 5.7. Future Directions

Several extensions follow directly from these limitations. First, pipeline synthesis should be repeated across independent fresh-context runs to quantify component and parameter selection consistency, processing order, and execution success. Such an analysis would distinguish reproducibility of the generated workflow from reproducibility of the underlying numerical operations.

Second, future evaluations should include larger datasets spanning different electrode systems, sampling rates, recording environments, and EEG paradigms. Testing across different datasets would make it possible to determine whether the orchestrator meaningfully adapts its choices to acquisition and task characteristics rather than producing a broadly similar workflow in most settings.

Third, evaluation should move beyond single-metric comparisons. A stronger validation framework would jointly assess data retention, residual artifact burden, preservation of known electrophysiological phenomena, spectral and temporal distortion, downstream feature stability, and computational cost. Where pipelines employ different filtering strategies, common-bandwidth analyses should be included prospectively. In addition, ablation analyses in which individual artifact processing operations are removed or varied while the remainder of the pipeline is held fixed, together with residual artifact measures, would allow the contribution of each artifact processing component to be isolated.

Fourth, future work could compare several decision architectures, including fully fixed pipelines, rule-based adaptive pipelines, LLM-directed pipelines, and hybrid systems in which numerical QC thresholds constrain the set of actions available to the orchestrator. This would help to identify which aspects of agentic reasoning provide measurable benefit beyond conventional automation.

Fifth, QC-driven adaptation should be evaluated more rigorously. Future studies could apply the framework to recordings with substantial artifact burden, such as extensive channel loss, high epoch rejection, or ICA failures, and could introduce quality control criteria that the orchestrator must evaluate and act upon; this would allow that the frequency and benefit of adaptive revisions to be measured directly.

Sixth, for downstream clinical tasks, future work could examine how performance changes when the orchestrator is exposed to more data. One approach is in-context learning under leave-one-subject-out validation, in which the orchestrator receives the labeled feature profiles of all remaining participants as reference examples before classifying each held-out participant.

Finally, the provenance mechanism provides an opportunity for additional human oversight. Rather than treating autonomy as an all-or-none property, future systems could allow investigators to inspect proposed operations, parameter rationales, QC results, and pipeline revisions before execution at predefined checkpoints. Such designs may be particularly useful in clinical and scientific settings where traceability and expert review are as important as automation.

### 5.8. Overall Interpretation

Taken together, the present findings support the feasibility of using an LLM-based orchestrator to synthesize executable EEG processing workflows from analytical objectives and a structured catalog of processing components. The principal contribution lies in separating task-directed pipeline orchestration from the underlying computational EEG operations while maintaining parameterization, structured execution feedback, and processing provenance.

The findings do not establish that agent-generated pipelines are globally optimal or universally superior to established EEG processing approaches; rather, they provide a foundation for further evaluating the reproducibility and scientific utility of agentic EEG pipeline synthesis. Establishing broader scientific utility will require evidence of repeatability across independent synthesis runs, generalization across heterogeneous EEG datasets and paradigms, and robust performance across different analytical objectives.

## 6. Conclusions

This study demonstrates the feasibility of using an LLM-based orchestrator to synthesize and execute task-specific EEG processing workflows through structured MCP-based tool interfaces. Rather than implementing the underlying numerical algorithms directly, the orchestrator selects, parameterizes, and orders EEG processing operations executed through EEGLAB/MATLAB within an architecture that supports quality control-informed adaptation. This architecture enables flexible workflow construction while maintaining explicit parameterization, quality control feedback, and execution provenance. In the present experiments, adaptation during execution consisted mainly of recovery from execution errors; while the architecture supports QC-driven revision of processing decisions, this has not yet been systematically demonstrated.

In the resting state evaluation, the generated workflow was executed across all eight recordings without manual modification of the processing sequence or parameter configuration. Relative to DISCOVER-EEG, it showed small mean differences in epoch retention and spectral entropy and higher SNR under the study’s specified evaluation procedure, although the SNR comparison was influenced by differences in filter boundaries. The downstream analysis consisting of Alzheimer’s disease classification versus healthy controls further demonstrated the feasibility of applying an agent-generated workflow to a clinically-oriented EEG biomarker task.

The proposed approach could be particularly useful for supporting investigators who need to construct complex EEG processing workflows while reducing the manual effort required to identify, configure, and connect individual processing components. By shifting part of this implementation burden to an agentic orchestration layer, the framework can allow researchers to devote more attention to scientific questions, evaluation, and interpretation. Future work should establish the repeatability and generalizability of the approach across larger datasets, independent synthesis runs, and a broader range of EEG applications, and should also evaluate QC-driven adaptation on recordings with substantial artifact burden. 

## Figures and Tables

**Table 1:** Representative principal components of the EEG processing catalog exposed to the agent through the MCP interface. For each component, the underlying EEGLAB/MATLAB (or FieldTrip) function, the parameters exposed to the orchestrator, and the output or quality control (QC) information returned are listed in order to support reproducibility and document the information available to the orchestrator for component selection, parameterization, compatibility checks, and quality control-informed adaptation. The complete component catalog exposed to the orchestrator includes components for source reconstruction, functional connectivity, graph-theoretic analysis, complexity analysis, aperiodic analysis, PREP, and NEAR, and is provided in Appendix A, Table A1. The current spectral analysis component uses a fixed 1–45 Hz range, while the DISCOVER-EEG comparison uses the 1–30 Hz evaluation range described in the Evaluation Metrics subsection.

Component	Underlying Function	Parameters Exposed to Agent	Output/QC Returned
**Data management**			
Load BIDS dataset	pop_importbids, pop_chanedit	Raw/output paths; study name; session, run, task labels; channel location mode; reference label and coordinates	STUDY created and per-recording .set saved; channel count and reference assignment summary
Load recording	eeg_checkset, pop_loadset	Recording index	Active EEG set; channel location backups initialized; channel count, sampling rate, sample count
Inspect dataset/state	EEG struct query (eeg_checkset)	None	Channel count, sampling rate, samples, trials, duration, ICA status/presence flag
Inspect events	EEG.event query	None	Event types and per-type counts
Preserve channel locations	Workspace assignment (EEG.urchanlocs, EEG_original_chanlocs)	None	Three channel location backups stored for later interpolation and re-referencing
**Signal preparation**			
Resample	pop_resample	Target sampling rate (Hz; default 128)	Updated sampling rate and sample count
Filtering (HP/LP/BP)	pop_eegfiltnew	High-pass and low-pass cutoffs (Hz); minimum-phase flag	Applied cutoffs; FIR order auto-computed; status
Line noise removal	pop_cleanline	Line frequencies (Hz); if unspecified, defaulted to the BIDS PowerLineFrequency value when available; additional harmonic frequencies could be specified explicitly by the orchestrator	Frequencies targeted for removal; execution status
Re-referencing	pop_reref	Reference channels (none = average reference); add-reference-channel flag	Reference scheme applied; channel count
**Artifact processing**			
Artifact cleaning/bad channel detection	pop_clean_rawdata (clean_rawdata/ASR)	Flatline, channel correlation, and line noise criteria; channels_only flag (default true); burst and window criteria when channels_only = false; internal high-pass band	Channel count before/after; number of channels removed; clean-channel-mask availability; artifact cleaning summary
ICA-based artifact correction	pop_runica (extended Infomax), pop_iclabel, pop_subcomp, pop_interp, pop_clean_rawdata	Number of ICA repetitions; per-category ICLabel rejection thresholds; burst and window criteria for the post-ICA artifact cleaning pass; post-ICA bad segment removal mode	Successful/failed ICA runs; representative-run selection summary; components classified and removed; post-ICA artifact cleaning summary; interpolation summary; final channel count
**Epoch processing**			
Segment continuous data	eeg_regepochs, pop_epoch	Epoch length; epoch overlap	Number of retained epochs and data shape; discontinuous epochs auto-rejected
Reject/interpolate contaminated epochs	pop_rejepoch, pop_interp	Voltage threshold (μV); maximum proportion of contaminated channels permitted before whole-epoch rejection	Number of channels interpolated and number of epochs rejected
**Analysis**			
PSD, band-power features & spectral entropy	pwelch (Hanning window)	Condition label; channels; Welch window length and overlap; spectral entropy flag	Absolute and relative band power (δ–γ), peak alpha frequency (IAF), spectral entropy; fixed 1–45 Hz analysis range; cached under condition label
**Output**			
Save processed dataset	pop_saveset	Output folder	Path to {original}_preprocessed.set

**Table 2:** Recording-level fidelity comparison between the MCP-mediated and original DISCOVER-EEG implementations.

Recording	Epochs	Mean Channel *r*	Global *r*	MAE (μV)	RMSE (μV)	Relative RMSE	Maximum Absolute Band-Power Difference
R1	301	1.0000	1.0000	0.0000	0.0000	0.00%	0.00%
R2	264	1.0000	1.0000	0.0000	0.0000	0.00%	0.00%
R3	278	1.0000	1.0000	0.0000	0.0000	0.00%	0.00%
R4	274	1.0000	1.0000	0.0000	0.0000	0.00%	0.00%
R5	283	1.0000	1.0000	0.0000	0.0000	0.00%	0.00%
R6	265	1.0000	1.0000	0.0000	0.0000	0.00%	0.00%
R7	264	1.0000	1.0000	0.0000	0.0000	0.00%	0.00%
R8	148	1.0000	1.0000	0.0000	0.0000	0.00%	0.00%

**Table 3:** Comparison of processing decisions made by the agent-generated and DISCOVER-EEG pipelines.

Processing Decision	Agent-Generated Pipeline	DISCOVER-EEG
Fundamental line noise removal	50 Hz	50 Hz
First-harmonic line noise removal	100 Hz	Not included
Frequency filtering	1–45 Hz band-pass	High-pass filtering; no corresponding 45 Hz upper cutoff
Pipeline construction	Synthesized from task specification and dataset information	Predefined reference workflow

**Table 4:** Epoch retention and spectral entropy comparisons between the agent-generated and DISCOVER-EEG pipelines. The Agent and DISCOVER columns report mean ± SD across the eight recordings, the paired difference column reports the mean within-recording difference ± SD of those differences, and the 95% CI is for the mean paired difference.

Metric	Agent	DISCOVER	Paired Difference	95% CI	Statistical Test
Epoch retention	0.845 ± 0.190	0.843 ± 0.153	0.002 ± 0.049	[−0.039, +0.044]	t(7)=0.143, p=0.891, $d_{z}=0.050$
Spectral entropy, 1–30 Hz	4.867 ± 0.332	4.841 ± 0.316	0.026 ± 0.056	[−0.021, +0.073]	t(7)=1.298, p=0.236, $d_{z}=0.459$

**Table 5:** Overall signal-to-noise ratio comparison between the agent-generated and DISCOVER-EEG pipelines. The Agent and DISCOVER columns report mean ± SD across the eight recordings, the paired-difference column reports the mean within-recording difference ± SD of those differences, and the 95% CI is for the mean paired difference.

Metric	Agent	DISCOVER	Paired Difference	95% CI	Statistical Test
Overall SNR	13.92 ± 2.91 dB	11.14 ± 2.84 dB	2.78 ± 0.49 dB	[+2.37, +3.19]	t(7)=16.15, p<0.001, $d_{z}=5.71$

**Table 6:** Band-specific signal-to-noise ratio comparisons between the agent-generated and DISCOVER-EEG pipelines. The Agent and DISCOVER columns report mean ± SD across the eight recordings, the paired-difference column reports the mean within-recording difference ± SD of those differences, and the 95% CI is for the mean paired difference.

Band	Agent	DISCOVER	Paired Difference	95% CI	Statistical Test
Delta	19.08 ± 1.81 dB	16.36 ± 1.68 dB	2.71 ± 0.56 dB	[+2.25, +3.18]	t(7)=13.78, p<0.001, $d_{z}=4.87$
Theta	14.90 ± 3.87 dB	12.18 ± 3.80 dB	2.72 ± 0.43 dB	[+2.36, +3.08]	t(7)=17.84, p<0.001, $d_{z}=6.31$
Alpha	15.37 ± 4.55 dB	12.52 ± 4.58 dB	2.85 ± 0.50 dB	[+2.43, +3.27]	t(7)=16.01, p<0.001, $d_{z}=5.66$
Beta	8.93 ± 2.25 dB	5.94 ± 2.39 dB	2.99 ± 0.50 dB	[+2.57, +3.41]	t(7)=16.96, p<0.001, $d_{z}=6.00$

**Table 7:** Confusion matrix for LLM-directed AD-versus-HC classification on the ds004504 dataset (36 AD, 29 HC), with AD treated as the positive class.

	Predicted AD	Predicted HC	Total
Actual AD	24	12	36
Actual HC	3	26	29
Total	27	38	65

**Table 8:** Classification performance for the AD-versus-HC task, with AD as the positive class.

Metric	Value
Accuracy	0.769
Precision	0.889
Recall (sensitivity)	0.667
Specificity	0.897
F1 score	0.762

**Table 9:** Feature vector used for the AD-versus-HC classification task. All features were channel-averaged; no channel-specific features were included.

Category	Features
Absolute band power ($\mu V^{2}$)	Delta, Theta, Alpha, Beta, Gamma, Total
Relative band power (%)	Delta, Theta, Alpha, Beta, Gamma
Peak frequency	IAF (7–13 Hz)
Spectral complexity	Shannon spectral entropy (bits)
Band ratios	θ/α, δ/α, α/θ, α/β, θ/β
Composite index	GSI =(δ+θ)/(α+β)
Reactivity	ARI $=\left(\alpha_{\mathrm{EC}}-\alpha_{\mathrm{EO}}\right)/\alpha_{\mathrm{EC}}$

## Data Availability

The EEG datasets analyzed in this study are publicly available through OpenNeuro. The resting state evaluation used the dataset described in the Methods, and the Alzheimer’s disease versus healthy control analysis used OpenNeuro datasets ds004504 and ds006036. Additional methodological information supporting reproduction of the analyses, including processing configurations and agent prompts, is provided in the manuscript and appendices.

## Abbreviations

The following abbreviations are used in this manuscript:

EEG	Electroencephalography
BCI	Brain–computer interface
LLM	Large language model
MCP	Model Context Protocol
ICA	Independent component analysis
ASR	Artifact subspace reconstruction
PSD	Power spectral density
SNR	Signal-to-noise ratio
BIDS	Brain Imaging Data Structure
AD	Alzheimer’s disease
HC	Healthy control
IAF	Individual alpha frequency
QC	Quality control
ERP	Event-related potential
PREP	Preprocessing Pipeline
HAPPE	Harvard Automated Processing Pipeline for Electroencephalography
FTD	Frontotemporal dementia
FFT	Fast Fourier transform
ROI	Region of interest
AEC	Amplitude-envelope correlation
dwPLI	Debiased weighted phase-lag index
MSE	Multiscale entropy
DFA	Detrended fluctuation analysis

## Appendix A. Complete EEG Processing Component Catalog

The catalog below lists all tools available to the orchestrator at the time of the experiment; inclusion in the catalog does not indicate that a tool was invoked in the evaluated pipeline.

**Table A1:** Complete catalog of EEG processing components exposed to the agent through the MCP interface. For each component (identified by its MCP tool name), the table lists the underlying EEGLAB/MATLAB, FieldTrip, or Python function, the parameters exposed to the orchestrator, and the returned output or quality control summary.

Component (MCP Tool)	Underlying Function	Parameters Exposed to Agent	Output/QC Returned
** Data management and input/output**			
load_bids_data	pop_importbids, pop_chanedit, pop_saveset	Raw/output paths; study name; session, run, task; channel location mode; reference label and coordinates; event marker	STUDY and per-recording .set saved; channel and reference assignment summary
load_bids_recording	eeg_checkset, pop_loadset	Recording index	Active EEG set; channel location backups initialised; channel count, sampling rate, samples
load_eeg_data	pop_loadeep_v, pop_chanedit	.cnt path; .elc montage; non-data channels	Active EEG set; montage captured; session initialised
load_set_file	pop_loadset	.set path	Preprocessed EEG loaded; ready for postprocessing
save_eeg	pop_saveset	Output folder	Path to {original}_preprocessed.set
reset_session	Session/MATLAB reset	None	Session cleared from memory
store_original_chanlocs	Workspace assignment	None	Three channel location backups stored for later interpolation/re-referencing
** Signal preparation**			
resample_eeg	pop_resample	Target sampling rate (Hz; default 128)	Updated sampling rate and sample count
filter_eeg	pop_eegfiltnew	High-pass and low-pass cutoffs (Hz); minimum-phase flag	Applied cutoffs; FIR order auto-computed; status
apply_cleanline	pop_cleanline	Line frequencies (Hz); if unspecified, defaults to BIDS PowerLineFrequency when available; additional harmonics may be specified	Frequencies targeted for removal; execution status
rereference_eeg	pop_reref	Reference channels (none = average); add-reference flag	Reference scheme applied; channel count
** Artifact and channel processing**			
clean_artifacts	pop_clean_rawdata (clean_rawdata/ASR)	Flatline, correlation, line noise, burst, window criteria; channels-only flag; internal high-pass	Channel count before/after; number removed; clean channel mask availability; seeded with the session base seed
run_ica	pop_runica, pop_iclabel, pop_icflag, pop_subcomp, pop_interp, pop_clean_rawdata	Repetitions; seven-category ICLabel thresholds; burst/window criteria; bad segment mode; single-thread flag	Successful/failed run counts; final channel count; representative run selection summary; per-repetition seed information
interpolate_missing_channels	pop_interp	Interpolation method (default spherical)	Channels restored to full montage; channel count
select_channels	pop_select	Channel indices or labels; exclude flag	Retained channel set; channel count
** Epoch and condition segmentation**			
epoch_resting_state	eeg_regepochs, pop_epoch	Epoch length; epoch overlap	Retained epochs and data shape; discontinuous epochs auto-rejected
epoch_eeg	pop_epoch	Event codes; time range (s)	Epoched dataset; number of epochs
epoch_eeg_for_ica	pop_epoch, eeg_epoch2continuous	Event marker; epoch bounds (s)	Task-relevant continuous data for ICA
baseline_correct	pop_rmbase	Baseline window (ms)	Baseline-corrected epochs
reject_bad_epochs	pop_rejepoch, pop_interp	Voltage threshold (μV); max bad channel fraction	Epochs rejected vs. channels interpolated counts
segment_by_condition	pop_select	Condition marker; photic keywords; edge trim; min block; mode	Kept time ranges; clean seconds; kept/photic/too-short block counts
** Spectral and band-power features**			
calculate_band_power	pwelch (Hanning window)	Condition label; channels; analysis window; Welch length/overlap; entropy flag	Absolute/relative band power (δ–γ); peak alpha (IAF); spectral entropy; cached
compute_power_fieldtrip	ft_freqanalysis (mtmfft), eeglab2fieldtrip	Frequency limits; taper; smoothing; frequency resolution; padding	Multitaper power spectrum in workspace and cached (DISCOVER-EEG-compatible)
compute_peakfrequency_fieldtrip	findpeaks + centre of gravity	Power label; alpha band	Local maximum and centre of gravity alpha peak frequency
calculate_ad_biomarkers	Python (from cached power)	Condition label	AD ratios (global slowing, θ/α, δ/α, …), relative power, IAF, entropy
calculate_alpha_reactivity	Python (from cached power)	None	Alpha reactivity index (eyes-closed vs. eyes-open)
compare_eyes_open_closed_alpha	Python (registry)	Eyes-open/closed labels	Band power, alpha ratio, peak alpha for both conditions
** Aperiodic (1/f) features**			
compute_aperiodic	specparam/FOOOF (Python)	Power label; fit range; aperiodic mode; peak limits; per-channel/average	Aperiodic exponent/offset/knee; periodic peaks; alpha peak; cached (optional JSON)
** Complexity and entropy features**			
compute_complexity_features	antropy (Python)	Condition label; channels; measures; max epochs; decimation	Permutation/sample entropy, Lempel–Ziv, fractal dimensions, DFA, Hjorth; cached
compute_multiscale_entropy	EntropyHub/built-in (Python)	Condition label; channels; scales; *m*; *r*; engine	MSE curve; complexity index; coarse-scale slope; cached
** Source reconstruction and connectivity**			
compute_source_lcmv	ft_prepare_sourcemodel, ft_prepare_leadfield, ft_timelockanalysis, ft_sourceanalysis (LCMV)	Band name/range; head model; atlas; template electrodes	Source struct and spatial filters in workspace (per band); DISCOVER-EEG-compatible
compute_connectivity_dwpli	ft_freqanalysis, ft_connectivityanalysis (wpli_debiased)	Band name/range; frequency resolution; smoothing; save path	Parcel-by-parcel dwPLI matrix; cached (requires ≥10 epochs)
compute_connectivity_aec	ft_virtualchannel, hilbert, aecConnectivity	Band name/range; save path	Orthogonalized parcel-by-parcel AEC matrix; cached
compute_graph_measures	Brain Connectivity Toolbox (degrees_und, clustering_coef_bu, charpath, efficiency_bin)	Band; measure (dwPLI/AEC); edge threshold	Degree, clustering, path length, global efficiency, small-worldness
** ROI biomarkers**			
classify_roi_anterior_posterior	Python (Schaefer atlas CSV)	Atlas path; anterior/posterior split	Anterior/posterior (and lobe) parcel index lists; cached
compute_source_band_power	MATLAB (source power averaging)	Band; ROI group; anterior/posterior indices	Mean source power over ROI (e.g., posterior beta)
** PREP pipeline**			
run_prep_pipeline	prepPipeline (PREP)	Line frequencies; reference/detrend type; thresholds; RANSAC toggle	Interpolated/still-noisy channels; per-method detections; robust reference applied
prep_robust_reference	performReference/robustReference (PREP)	Reference and mean-estimate type; thresholds; RANSAC toggle	Robust average reference; diagnostics in noiseDetection
prep_find_noisy_channels	findNoisyChannels (PREP)	Thresholds; RANSAC parameters; detrend	Per-channel noise metrics; flagged channels (no data modification)
prep_remove_trend	removeTrend (PREP)	Detrend type; cutoff; step size	Detrended signal (in place)
get_prep_noise_detection	EEG.etc.noiseDetection query	None	PREP status; interpolated/still-noisy channels; per-method breakdown; version
** NEAR bad-channel pipeline**			
near_detect_bad_channels	NEAR_getBadChannels, pop_select	Flatline/LOF/periodogram options; population; remove flag	Per-method bad channels; LOF vector; channel count before/after
** Quality control and status**			
get_eeg_info	EEG struct query	None	Channel count, sampling rate, samples, trials, duration, ICA flag
get_eeg_snapshot	MATLAB (data slice)	Number of channels; number of samples	Matrix dimensions and numeric preview; NaN check
get_event_info	EEG.event query	None	Event types and counts
get_clean_channel_mask	EEG.etc query	None	Boolean kept/removed channel mask
check_ica_status	EEG struct query	None	Whether ICA and ICLabel have completed
inspect_event_timeline	EEG.event query	Photic keywords; max events	Event onsets (s); inter-marker blocks with photic overlap flags
assess_vigilance_state	pwelch (MATLAB)	Posterior/anterior channels; time bins; alpha/theta bands	Posterior alpha peak, anteriorisation, θ/α, alpha time course; non-diagnostic impression
** Reproducibility and random initialization**			
set_random_seed	set_random_seed (custom MATLAB; RandStream mrg32k3a)	Base seed (integer; default 42) or none for unseeded, time-based initialization	Session base seed set; downstream stochastic steps (channel rejection, ICA repetitions, graph null models) derive step-specific seeds
get_random_seed	Session query	None	Current base seed and seeding status
** Result management and knowledge retrieval**			
get_freq_analysis_results	In-memory registry	Condition label	Cached band-power results (or summary of all)
clear_freq_analysis_results	In-memory registry	None	Frequency-result cache cleared
get_aperiodic_results	In-memory registry	Output label	Cached aperiodic fit
clear_aperiodic_results	In-memory registry	Output label	Aperiodic cache cleared
get_complexity_results	In-memory registry	Condition label	Cached complexity/MSE results
clear_complexity_results	In-memory registry	None	Complexity cache cleared
get_power_results	In-memory registry	Output label	Cached FieldTrip power-spectrum metadata
clear_power_results	In-memory registry + MATLAB	None	Power cache and workspace variables cleared
get_connectivity_results	In-memory registry	None	Stored source filters and connectivity matrices (metadata)
clear_connectivity_results	In-memory registry + MATLAB	None	Filters/matrices and workspace variables cleared
save_subject_features	CSV append (host)	Subject ID; ground-truth/clinical labels; output path; condition label	Row appended to eeg_features.csv (header created if absent)
load_subject_features	CSV read (host)	Output path; subject ID; class filter	Feature rows and group-level (AD/HC) summary statistics
eeg_rag	RAG vector store (Python)	Query	Relevant EEG-knowledge passages

## Appendix B. Agent Task Prompts

### Appendix B.1. Agentic Pipeline Synthesis and Quality (RQ2–RQ3)

The verbatim task prompt provided to the orchestrator is reproduced below.

You are an autonomous EEG preprocessing agent that operates exclusively using tools provided by the MCP server (basic-eeg-preprocessing-mcp). Figure out the optimal pipeline based on the MCP server tools to preprocess resting-state EEG Data. The pipeline will accept resting-state EEG data from Germany organized according to the Brain Imaging Data Structure (BIDS) standard where the bids task label should be empty. The pipeline will automatically extract physiologically meaningful EEG features known to be relevant in neuropsychiatric conditions, specifically: oscillatory power spectra and alpha peak frequency in electrode space, and functional connectivity (both phase-based and amplitude-based) alongside graph-theoretic brain network measures in source space. State the determined pipeline concisely in steps.

### Appendix B.2. EEG Pipeline Synthesis for Downstream Clinical Reasoning (RQ4)

The task prompt provided to the orchestrator is reproduced below. Local file paths have been replaced with bracketed placeholders, and the participant number and recording index (shown here as #) were filled in for each participant.

You are an autonomous EEG clinical analysis agent operating through the basic-eeg-preprocessing-mcp MCP server. A patient has arrived at a neurology clinic for cognitive assessment. Resting-state EEG recordings have been acquired during both Eyes Closed and Eyes Open conditions. Your task is to perform a clinically informed EEG evaluation. Before performing any analysis, use the available MCP tools to discover what preprocessing, feature extraction, biomarker calculation, and analysis capabilities are available. Based on the available tools, determine and execute the most appropriate EEG preprocessing pipeline and analysis workflow.
**Patient EEG Data Locations**
Raw Eyes Closed EEG: [raw eyes-closed BIDS directory] Raw Eyes Open EEG: [raw eyes-open BIDS directory] Processed Eyes Closed EEG: [processed eyes-closed output directory] Processed Eyes Open EEG: [processed eyes-open output directory] Saved Results: [results output directory]
**Clinical Context**
The EEG recordings were acquired at the 2nd Department of Neurology of AHEPA General Hospital of Thessaloniki using a Nihon Kohden EEG-2100 system with 19 scalp electrodes positioned according to the International 10–20 System. Recordings were originally acquired using Cz as the hardware reference and were subsequently re-referenced by the recording system to linked mastoids (A1–A2). BIDS metadata confirms: EEGReference = “A1 A2”
**Eyes Open Protocol**
During the Eyes Open recordings, participants remained awake with their eyes open while receiving intermittent photic stimulation. Stimulation frequencies included 5 Hz, 10 Hz, 15 Hz, and in some recordings extended up to 30 Hz in 5 Hz increments. Account for these stimulation conditions when interpreting spectral findings.
**BIDS Task Labels**
Eyes Closed recordings: task = “eyesclosed” Eyes Open recordings: task = “photomark”
**Clinical Objective**
Analyze both Eyes Closed and Eyes Open EEG recordings for the patient.

1. Determine and execute an appropriate preprocessing pipeline.
2. Evaluate data quality and artifact removal effectiveness.
3. Extract relevant EEG biomarkers, and quantitative metrics.
4. Compare Eyes Closed and Eyes Open findings.
5. Integrate all quantitative findings into a clinical interpretation. The clinical diagnosis must be based on the combined evidence from both Eyes Closed and Eyes Open EEG recordings and justified using explicit clinical reasoning grounded in the calculated EEG biomarkers.

Analyze only Patient #. Recording_index: # Please do the EO phase first. Only load the bids data once.
